# Urinary concentrations of GHB and its novel amino acid and carnitine conjugates following controlled GHB administration to humans

**DOI:** 10.1038/s41598-023-36213-1

**Published:** 2023-06-02

**Authors:** Andrea E. Steuer, Francesco Bavato, Laura K. Schnider, Dario A. Dornbierer, Oliver G. Bosch, Boris B. Quednow, Erich Seifritz, Christian Steuer, Thomas Kraemer

**Affiliations:** 1 Department of Forensic Pharmacology and Toxicology, Zurich Institute of Forensic Medicine, University of Zurich, Winterthurerstrasse 190/52, 8057 Zurich, Switzerland; 2 Department of Psychiatry, Psychotherapy, and Psychosomatics, Psychiatric University Hospital Zurich, University of Zurich, 8032 Zurich, Switzerland; 3 Neuroscience Center Zurich, University of Zurich and Swiss Federal Institute of Technology Zurich, 8057 Zurich, Switzerland; 4 Institute of Pharmaceutical Sciences, Swiss Federal Institute of Technology Zurich, 8093 Zurich, Switzerland

**Keywords:** Biomarkers, Analytical chemistry

## Abstract

Gamma-hydroxybutyrate (GHB) remains a challenging clinical/forensic toxicology drug. Its rapid elimination to endogenous levels mainly causes this. Especially in drug-facilitated sexual assaults, sample collection often occurs later than the detection window for GHB. We aimed to investigate new GHB conjugates with amino acids (AA), fatty acids, and its organic acid metabolites for their suitability as ingestion/application markers in urine following controlled GHB administration to humans. We used LC–MS/MS for validated quantification of human urine samples collected within two randomized, double-blinded, placebo-controlled crossover studies (GHB 50 mg/kg, 79 participants) at approximately 4.5, 8, 11, and 28 h after intake. We found significant differences (placebo vs. GHB) for all but two analytes at 4.5 h. Eleven hours post GHB administration, GHB, GHB-AAs, 3,4-dihydroxybutyric acid, and glycolic acid still showed significantly higher concentrations; at 28 h only GHB-glycine. Three different discrimination strategies were evaluated: (a) GHB-glycine cut-off concentration (1 µg/mL), (b) metabolite ratios of GHB-glycine/GHB (2.5), and (c) elevation threshold between two urine samples (> 5). Sensitivities were 0.1, 0.3, or 0.5, respectively. Only GHB-glycine showed prolonged detection over GHB, mainly when compared to a second time- and subject-matched urine sample (strategy c).

## Introduction

**Figure 1:**
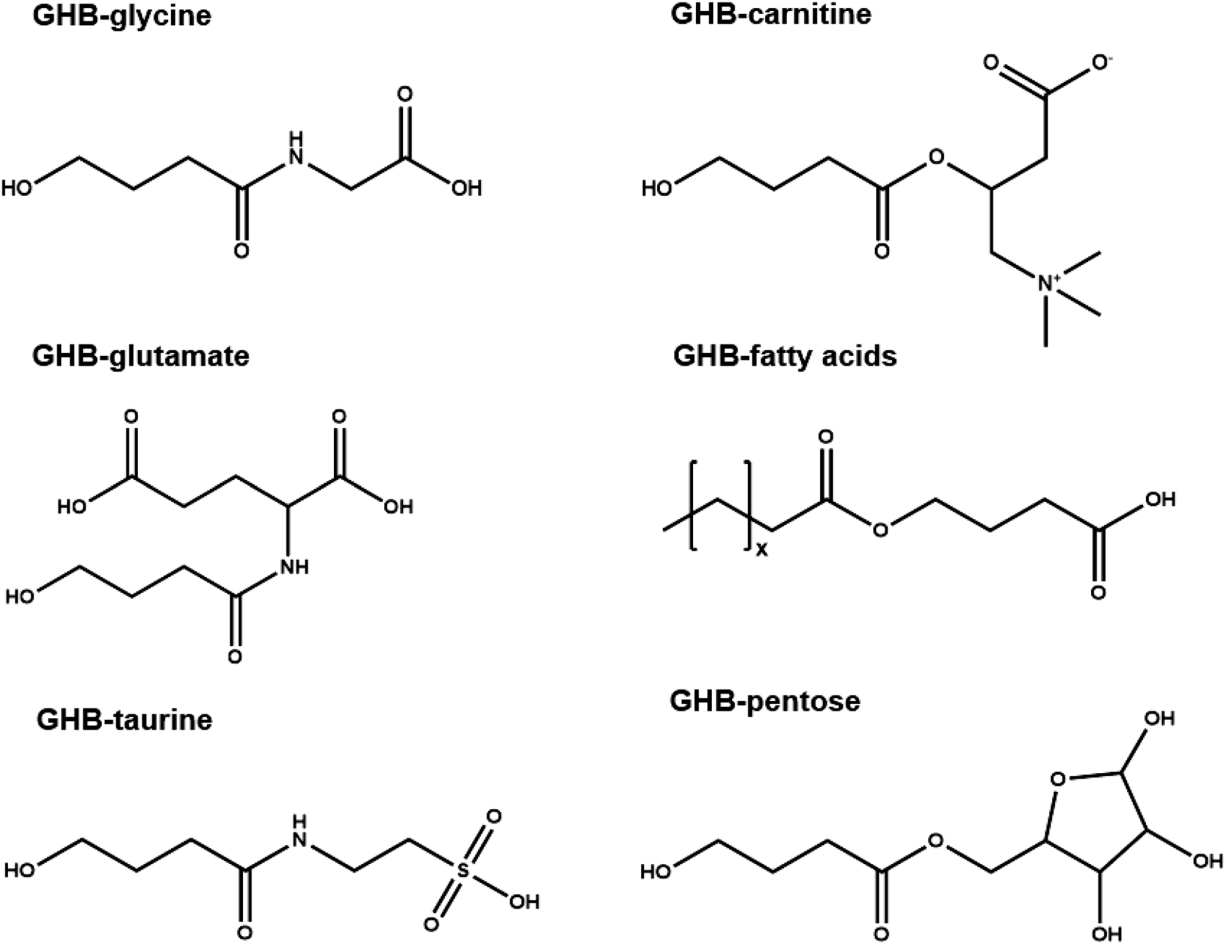
Chemical structures of newly identified GHB conjugates with amino acids glycine, glutamate, and taurine (left), carnitine, fatty acids, or pentose (right).

Gamma-hydroxybutyrate (GHB), a short-chain fatty acid, represents an important analyte in clinical and forensic toxicology not only because of its recreational consumption as a drug of abuse (DOA) but also because of its use in drug-facilitated crimes or drug-facilitated sexual assaults (DFSA)^1–3^. Matrices such as blood or urine only allow short detection windows for GHB up to 6 h and 12 h, respectively^4^. To make matters worse, GHB is also an endogenous compound^5,6^, which makes it especially challenging to discriminate low exogenous GHB levels following GHB consumption/administration from endogenous levels. Cut-offs between 6 and 10 µg/mL are commonly recommended in urine to differentiate between endogenous GHB levels and GHB intake^1,5,7^. Still, additional biomarkers are required to improve GHB detection and interpretation over longer intervals. GHB-glucuronide and GHB-sulfate were extensively investigated. While controversially discussed, most studies found no advantages in detecting GHB-glucuronide^8–11^. More recently, organic acids formed through GHB degradation or beta-oxidation gained attention as additional biomarkers. Endogenous concentrations of 2,4-dihydroxybutyric acid (2,4-DHB), 3,4-DHB, glycolic acid (GA), succinic acid (SA), and succinylcarnitine were systematically determined in larger cohorts^12,13^, and their general usefulness in improving GHB detection windows and interpretation was demonstrated^14–16^. New conjugates of GHB (Fig. 1) with carnitine, amino acids (glycine, glutamate, taurine, phenylalanine), pentose, fatty acids, phospholipids, and some still unknown features (U3, U4, U16) also pointed towards promising additional GHB markers. They were discovered either through untargeted metabolic profiling^17,18^ or hypothesis-driven approaches^19–21^. Systematic studies on their urinary concentrations are still pending, though. Therefore, we aimed to quantitatively characterize urinary excretion of GHB, GHB-carnitine, GHB-glycine, GHB-glutamate, GHB-taurine, GHB-phenylalanine, GHB-fatty acid esters (C8–C18, C18:1) and organic acids 2,4-DHB, 3,4-DHB, GA, SA, succinylcarnitine after controlled administration of GHB to humans and to assess their usefulness for prolonged detection of GHB intake/application applying three different discrimination strategies.

## Results

### Study cohorts, analytics, and measured concentrations

**Figure 2:**
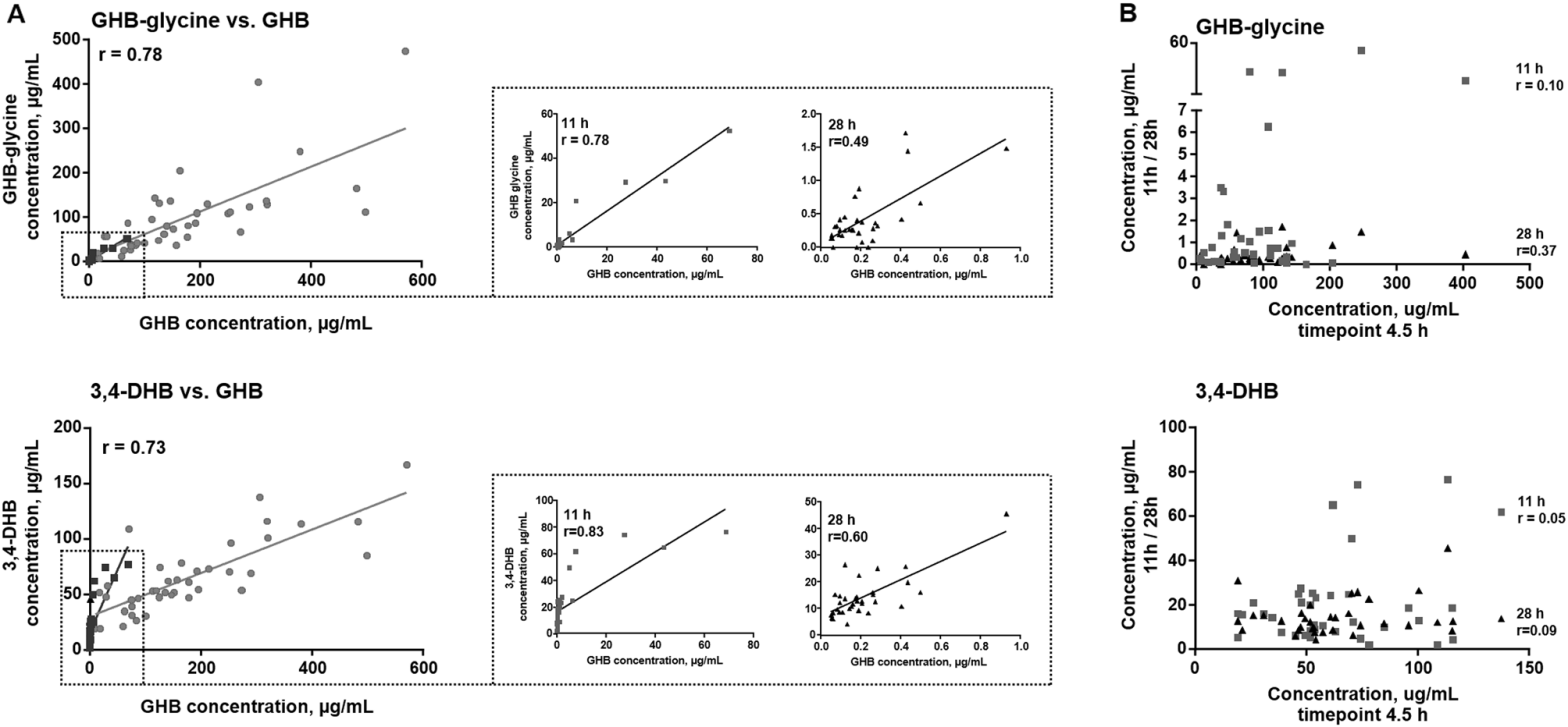
Correlation analysis (spearman, r) between (**A**) urinary GHB concentration (x-axis) and GHB-glycine or 3,4-DHB concentrations at 4.5 h (light grey), 11 h (dark grey), and 28 h (black) after GHB intake and (**B**) correlation between concentrations at 4.5 h versus those at 11 h (dark grey) and 28 h (black). Solid lines represent results for linear correlation.

**Figure 3:**
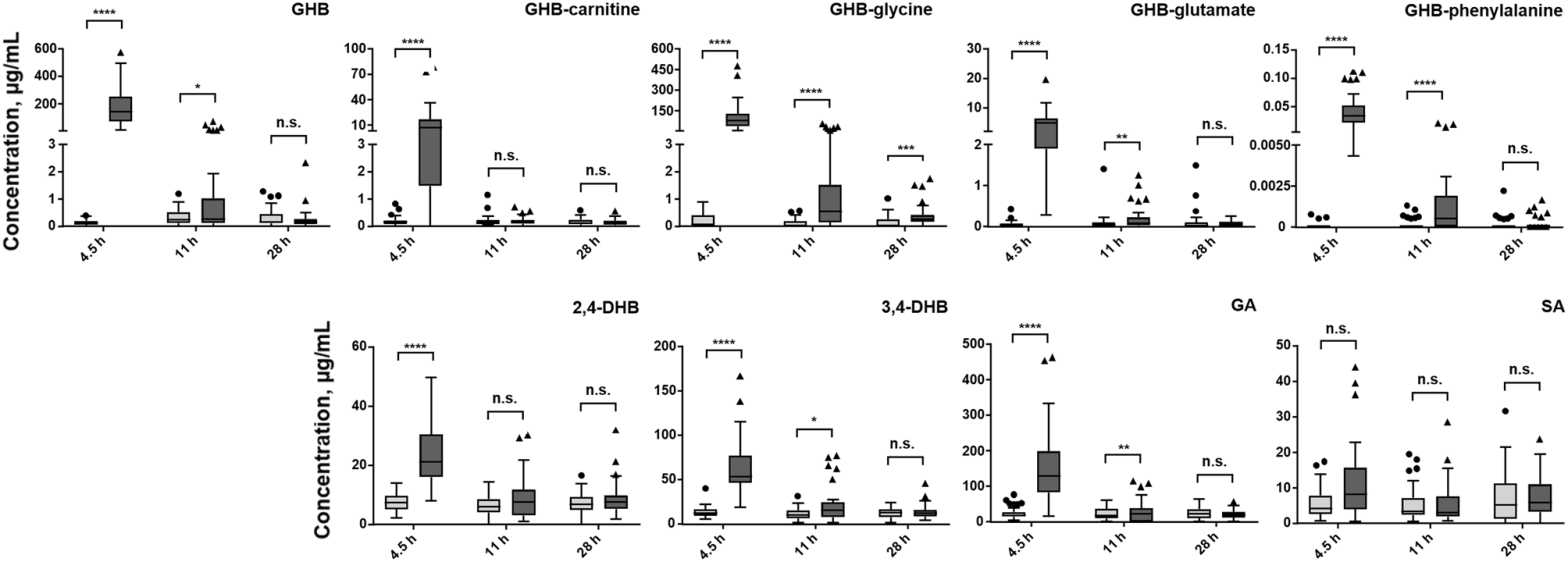
Box plots of urine concentrations for placebo (light grey) and GHB treatment (dark grey) collected in study II at 4.5, 11, and 28 h after intake. Statistical comparison was performed using a one-tailed, nonparametric Mann–Whitney test (p < 0.05): *p < 0.05; **p < 0.01; ***p < 0.001; ****p < 0.0001.

Each participant's concentrations determined using a validated liquid chromatography—tandem mass spectrometry (LC–MS/MS) method^21^ and urinary creatinine are provided in Table S1. Comparison of calculated concentrations between ^12^C- and ^13^C-isotopes indicated good agreement within the calibration range but a poorer agreement for ^12^C in higher concentrations (Supplementary Fig. S1). Consequently, ^13^C-results were used as approximate values for GHB concentrations in samples exceeding calibration. Table 1 provides a summary (mean, median, range) of the concentrations determined for each condition un-/adjusted to creatinine, respectively. Creatinine concentrations were found to be independent of treatment, gender, or disease but showed expected intra-day variation (study II, 11 h, Supplementary Fig. S2). GHB-carnitine and GHB-amino acid conjugates were detected in both placebo and GHB sessions. Only GHB-taurine concentrations were below the limit of quantification (LOQ) of 0.05 µg/mL in all placebo samples. GHB fatty acid conjugates could not be detected in any treatment condition. As expected, the highest concentrations of all measured compounds were observed in the earliest urine sample collected in the GHB session. Compared to a limited number of authentic cases published in former studies (ForTox pos 1^21^, ForTox pos 2^15^, n = 7 each, Table 1), concentrations determined after controlled administrations (4.5 h) were lower for all compounds except for GHB-carnitine. Spearman correlation between unadjusted and creatinine-adjusted concentrations was high (r > 0.8). Only DHB isomers and GHB-glucuronide (r 0.4–0.6), SA, and succinylcarnitine (r 0.6–0.7) showed poor(er) correlation (Supplementary Fig. S3). Comparison between 4.5 h samples from cohorts I and II revealed no relevant differences, except for GHB-carnitine, where significantly higher concentrations were measured in study cohort I. Amino acid conjugate concentrations were slightly higher in cohort II. Concentrations were independent of age, sex, and underlying depressive disorder. Only SA concentrations were significantly higher in females at any timepoint and 2,4-DHB in depressive patients at 4.5 h (Supplementary Fig. S4). We found endogenous concentrations of GHB and GHB-metabolites (placebo) to be independent of daytime, except for GHB-glucuronide and succinylcarnitine, which showed significantly lower concentrations in the afternoon (Fig. 3, Supplementary Fig. S5). Spearman correlation of metabolite concentrations to those of GHB in study cohort II was high at 4.5 h and 11 h and moderate at 28 h, as shown in representative examples in Fig. 2A. In contrast, initial concentration did not influence concentrations at later time points, as indicated by a lack of correlation between early urine concentrations (4.5 h) and those at 11 h or 28 h, respectively (Fig. 2B).

**Table 1:** Measured urinary concentrations of GHB and potential biomarkers in µg/mL (range, mean, median), unadjusted and normalized to 100 mg/dL creatinine. Treatment groups included placebo and GHB in study cohort I (4.5 h, n = 19 each; 8 h, n = 17, n = 18, respectively) and study cohort II (4.5 h, n = 40 each; 11 h, n = 35, n = 37, respectively; 28 h, n = 34, n = 35, respectively). Sample numbers (percentage) outside the calibration are indicated when exceeding calibration (> Cal8); detected but below the lowest calibrator (< LOQ); or non-detected (n.d.). Additionally, concentrations determined in different authentic forensic toxicology cases in former studies (ForTox neg 1^21^; ForTox neg 2^12^) or a clinical study (ClinStudy^13^) unrelated to GHB, as well as from few forensic GHB-positive cases (n = 7, each) ForTox pos 1^21^ and ForTox pos 2^15^ are added for comparison purposes only.

	Study cohort	Time point	Concentration, µg/mL (unadjusted)			Samples outside the calibration range			Concentration µg/mL (normalized to 100 mg/dL)		
			Range	Mean	Median	> Cal 8, %	< LOQ, %	n.d., %	Range	Mean	Median
**GHB**											
Placebo	I	4.5 h	< LOQ–0.41	0.20	0.20		5		< LOQ–0.35	0.12	0.11
	I	8 h	< LOQ–0.35	0.16	0.14		11		< LOQ–0.17	0.07	0.06
	II	4.5 h	< LOQ–0.39	0.17	0.14		10		< LOQ–0.32	0.12	0.09
	II	11 h	< LOQ–1.2	0.36	0.27		6		< LOQ–2.5	0.52	0.28
	II	28 h	< LOQ–1.3	0.36	0.20		15		< LOQ–1.28	0.34	0.16
ForTox neg 1			0.05–2.3	0.95	0.56				0.14–4.1	1.0	0.47
GHB	I	4.5 h	58–240	120	110	63			25.5–130	75	67
	II	4.5 h	8.8–570	180	140	70			4.9–480	140	130
	I	8 h	1.4–76	33	30				2.0–42	17	18
	II	11 h	< LOQ–69	5.1	0.37		8		< LOQ–39	3.6	0.73
	II	28 h	< LOQ–2.3	0.27	0.18		3		< LOQ–1.9	0.23	0.13
ForTox pos 1			280–3500	1200	850	100			110–2100	710	360
**GHB-carnitine**											
Placebo	I	4.5 h	n.d.–0.46	0.22	0.16			16	n.d.–0.26	0.12	0.13
	I	8 h	n.d.–0.39	0.17	0.19			11	n.d.–0.30	0.09	0.09
	II	4.5 h	n.d.–0.81	0.20	0.17			10	n.d.–0.29	0.12	0.10
	II	11 h	0.05–1.15	0.21	0.14				0.07–0.92	0.27	0.21
	II	28 h	n.d.–0.59	0.19	0.14		3	9	n.d.–0.63	0.18	0.15
ForTox neg 1			n.d.–1.0	0.32	0.23			50	n.d.–0.38	0.20	0.21
GHB	I	4.5 h	1.5–670	160	94	47			1.3–390	96	64
	II	4.5 h	n.d.–78	13	7.0			3	n.d.–59	10	6
	I	8 h	n.d.–120	22	13			6	n.d.–40	11	7
	II	11 h	n.d.–0.68	0.21	0.17		5	3	n.d.–1.1	0.31	0.24
	II	28 h	n.d.–0.54	0.18	0.15		6	17	n.d.–0.22	0.10	0.10
ForTox pos 1			5.1–350	120	120	14			5.7–150	48	37
**GHB-glycine**											
Placebo	I	4.5 h	n.d.–0.26	0.12	0.10		5	21	n.d.–0.13	0.06	0.06
	I	8 h	n.d.–0.35	0.08	0.09		6	28	n.d.–0.10	0.04	0.04
	II	4.5 h	n.d.–0.89	0.36	0.34		0	45	n.d.–0.53	0.13	0.09
	II	11 h	n.d.–0.56	0.26	0.25		3	51	n.d.–0.90	0.14	0.00
	II	28 h	n.d.–1.0	0.35	0.27		3	50	n.d.–0.49	0.12	0.00
ForTox neg 1			n.d.–0.81	0.36	0.36			33	n.d.–1.1	0.35	0.20
GHB	I	4.5 h	11–140	55	44				5.4–80	33	31
	II	4.5 h	6.2–470	100	81				3.4–240	82	51
	I	8 h	0.79–35	17	17				0.79–17	8.6	9.3
	II	11 h	n.d.–52	4.5	0.56			3	n.d.–29	3.3	1.0
	II	28 h	n.d.–1.7	0.45	0.31		3	9	n.d.–0.62	0.28	0.25
ForTox pos 1			130–1700	600	420	43			50–640	320	200
**GHB-glutamate**											
Placebo	I	4.5 h	n.d.–0.13	0.01	0.00		16	79	n.d.–0.10	0.01	0.00
	I	8 h	n.d.–0.07	0.01	0.00		6	83	n.d.–0.09	0.01	0.00
	II	4.5 h	n.d.–0.43	0.11	0.08		18	33	n.d.–0.58	0.06	0.03
	II	11 h	n.d.–1.40	0.18	0.10		29	17	n.d.–0.79	0.14	0.10
	II	28 h	n.d.–1.48	0.24	0.10		18	32	n.d.–0.88	0.10	0.05
ForTox neg 1			n.d.–< LOQ				67	33	n.d.–< LOQ		
GHB	I	4.5 h	1.2–5.9	2.6	2.2				0.59–2.9	1.5	1.3
	II	4.5 h	0.29–20	4.9	4.7				0.16–15	4.0	3.3
	I	8 h	n.d.–1.8	0.81	0.80		6	6	n.d.–0.96	0.43	0.45
	II	11 h	n.d.–1.2	0.26	0.15		22	3	n.d.–0.95	0.29	0.23
	II	28 h	n.d.–0.26	0.11	0.09		14	14	n.d. 0.28	0.07	0.07
ForTox pos 1			17–110	52	43	14			6.5–63	29	18
**GHB-phenylalanine**											
Placebo	I	4.5 h	< LOQ–< LOQ				100	0	< LOQ–< LOQ		
	I	8 h	n.d.–< LOQ				83	17	n.d.–< LOQ		
	II	4.5 h	n.d.–0.0008	0.0006	0.0006		53	40	n.d.–0.0008	0.0001	0.0000
	II	11 h	n.d.–0.0013	0.0008	0.0006		49	31	n.d.–0.0039	0.0005	0.0000
	II	28 h	n.d.–0.0022	0.0009	0.0006		44	38	n.d.–0.0027	0.0003	0.0000
ForTox neg 1			n.d.–< LOQ				50	50	n.d.–< LOQ		
GHB	I	4.5 h	0.016–0.062	0.031	0.027				0.0084–0.039	0.019	0.017
	II	4.5 h	0.0044–0.11	0.040	0.034	5			0.0024–0.095	0.032	0.028
	I	8 h	0.0013–0.017	0.0091	0.011				0.0007–0.011	0.0051	0.0051
	II	11 h	< LOQ–0.019	0.0035	0.0011		43		< LOQ–0.011	0.0027	0.0015
	II	28 h	n.d.–0.0016	0.0010	0.0009		69	14	n.d.–0.0019	0.0005	0.0004
ForTox pos 1			0.023–0.90	0.32	0.18	57			0.0084–0.46	0.17	0.13
**GHB-taurine**											
Placebo	I	4.5 h	n.d.–< LOQ				100		n.d.–< LOQ		
	I	8 h	n.d.–< LOQ				44	56	n.d.–< LOQ		
	II	4.5 h	n.d.–< LOQ				80	20	n.d.–< LOQ		
	II	11 h	n.d.–< LOQ				69	31	n.d.–< LOQ		
	II	28 h	n.d.–< LOQ				76	24	n.d.–< LOQ		
ForTox neg 1			n.d.–< LOQ				67	33	n.d.–< LOQ		
GHB	I	4.5 h	0.05–0.59	0.19	0.12				0.03–0.33	0.11	0.10
	II	4.5 h	< LOQ–3.0	0.53	0.32		8		< LOQ–1.1	0.36	0.27
	I	8 h	< LOQ–0.15	0.10	0.08		39		< LOQ–0.06	0.04	0.04
	II	11 h	< LOQ–0.23	0.13	0.11		89		< LOQ–0.13	0.07	0.07
	II	28 h	< LOQ–< LOQ				100		< LOQ–< LOQ		
ForTox pos 1			1.2–12	4.1	2.7				0.37–5.1	2.0	1.4
**GHB-glucuronide**											
Placebo	I	4.5 h	0.21–1.8	0.92	0.75				0.21–0.73	0.48	0.46
	I	8 h	0.26–2.9	1.2	1.1				0.22–1.1	0.58	0.52
	II	4.5 h	0.12–2.1	0.68	0.55				0.11–1.4	0.48	0.38
	II	11 h	< LOQ–1.0	0.39	0.26		6		< LOQ – 2.4	0.58	0.43
	II	28 h	0.07–1.6	0.5	0.48				0.13 – 2.3	0.54	0.48
ForTox neg 1			0.37–11	4.1	2.9				1.1 – 7.5	3.2	2.8
GHB	I	4.5 h	0.69–3.2	1.6	1.5				0.47 – 2.6	0.96	0.75
	II	4.5 h	0.10–2.9	1.1	1.0				0.06 – 1.7	0.88	0.90
	I	8 h	0.20–3.3	1.6	1.5				0.25–1.5	0.81	0.78
	II	11 h	0.08–1.3	0.37	0.31				0.07 – 5.1	0.96	0.51
	II	28 h	0.11–1.8	0.63	0.55				0.07–1.4	0.51	0.37
ForTox pos 1			4.8–14	8.6	7.8				2.1–18	5.3	3.2
**2,4 DHB**											
Placebo	I	4.5 h	2.4–20	11	11				2.9–9.4	6.1	6.4
	I	8 h	2.9–21	8.6	7.8				2.4–8.3	4.3	3.6
	II	4.5 h	2.3–14	7.7	7.5				2.5–13	5.4	5.2
	II	11 h	< LOQ–14	6.6	6.1		3		< LOQ–21	8.5	6.9
	II	28 h	< LOQ–17	7.5	6.8		6		< LOQ–14	6.1	5.2
ForTox neg 1			0.94–22	12	13				5.6–15	9.9	10.0
ForTox neg 2			0.72–26								
ClinStudy			0.76–36								
GHB	I	4.5 h	15–38	24	23	16			7.7–34	15	14
	II	4.5 h	8.0–50	24	21	25			4.4–32	19	17
	I	8 h	4.9–29	17	19	0			5.2–13	9.2	9.0
	II	11 h	1.1–30	9.2	7.7	3			4.2–33	13	12
	II	28 h	1.9–32	8.7	7.6	3			2.5–26	6.9	6.2
ForTox pos 1			31–140	78	72	86			13.2–104	40	38
ForTox pos 2			3.8–319								
**3,4 DHB**											
Placebo	I	4.5 h	3.7–40	17	16				4.4–14	10	10
	I	8 h	6.0–31	14	13				4.6–12	7.2	6.4
	II	4.5 h	5.6–40	14	13				4.7–18	10	9.1
	II	11 h	1.6–31	11	10				6.0–35	15	13
	II	28 h	1.7–24	12	13				4.0–25	11	9.1
ForTox neg 1			2.1–36	18	17				6.7–25	16	16
ForTox neg 2			1.9–120								
ClinStudy			0.66–53								
GHB	I	4.5 h	34–74	51	49	42			17–55	31	30
	II	4.5 h	19–70	65	54	65			10–140	52	46
	I	8 h	8.9–59	40	40	28			9.9–46	22	20
	II	11 h	2.1–77	21	16	11			5.9–69	29	27
	II	28 h	4.2–46	14	13				5.0–26	11	11
ForTox pos 1			71–160	123	121	100			26–170	75	47
ForTox pos 2			6.3–850								
**GA**											
Placebo	I	4.5 h	16–160	62	62				17–78	35	34
	I	8 h	22–110	57	57				15–42	28	30
	II	4.5 h	5.4–76	24	19				5.4–34	16	15
	II	11 h	< LOQ–61	24	19		6		< LOQ–100	29	23
	II	28 h	< LOQ–64	27	24		9		< LOQ–53	20	18
ForTox neg 1			< LOQ–110	61	67		17		< LOQ–65	37	34
ForTox neg 2			1.3–400								
ClinStudy			5.1–210								
GHB	I	4.5 h	97–310	180	170				46–240	110	100
	II	4.5 h	17–460	160	130	5			13–440	130	110
	I	8 h	19–280	120	110				32–96	59	58
	II	11 h	n.d.–110	42	34		32	3	n.d.–63	34	32
	II	28 h	< LOQ–55	22	20		3		< LOQ–57	17	15
ForTox pos 1			379–1800	860	600	86			140–940	440	390
ForTox pos 2			19–1800								
**SA**											
Placebo	I	4.5 h	1.8–11	5.1	4.8		5		1.1–8.5	2.9	2.3
	I	8 h	0.75–8.9	4.4	3.5				0.75–6.8	2.2	1.7
	II	4.5 h	0.71–17	5.6	4.3				0.63–13	3.9	3.4
	II	11 h	0.64–19	5.6	3.4				0.34–20	7.6	7.9
	II	28 h	0.89–32	8.4	7.1		9		0.63–13	3.9	3.4
ForTox neg 1			1.3–61	23	17		17		2.2–42	13	7
ForTox neg 2			1.2–2.7								
ClinStudy			0.20–13								
GHB	I	4.5 h	2.8–32	13	11				2.0–18	7.8	6.4
	II	4.5 h	0.66–44	11	8.2				0.42–27	8.6	6.8
	I	8 h	1.4–19	8.5	7.3				1.5–11	4.8	4.5
	II	11 h	0.72–28	5.7	3.1				0.60–35	9.5	6.7
	II	28 h	0.82–24	7.4	6.0		3		0.71–34	6.3	5.6
ForTox pos 1			28–110	54	47	29			12.5–52	27	19
ForTox pos 2			< LOD–7.0								
**Succinylcarnitine**											
Placebo	I	4.5 h	0.19–26	4.0	1.8				0.23–10	1.8	1.2
	I	8 h	0.85–13	3.9	2.5				0.61–3.8	1.8	1.6
	II	4.5 h	0.42–7.4	2.4	1.6				0.32–10	2.0	1.0
	II	11 h	0.13–4.4	0.9	0.5				0.11–8.0	1.4	0.80
	II	28 h	0.19–7.6	1.7	1.2				0.17–5.6	1.5	0.96
ForTox neg 1			0.44–14	4.5	1.8				1.1–5	3	3
ClinStudy			0.10–4.3								
GHB	I	4.5 h	1.1–13	3.0	2.2				0.64–6.1	1.8	1.3
	II	4.5 h	0.31–10	2.5	1.6				0.20–10	2.1	1.6
	I	8 h	0.73–9.4	3.9	3.1				0.54–4.0	1.9	1.5
	II	11 h	0.15–3.5	0.7	0.6				0.15–15	2.0	0.84
	II	28 h	0.28–7.4	2.2	1.4				0.33–8.9	2.0	1.1
ForTox pos 1			1.4–34	12	9.7				1.9–9.2	4.6	3.6

### Differentiation based on concentrations/cut-off values (Strategy a)

Concentrations derived from placebo and GHB sessions from study cohort II at 4.5, 11, and 28 h after intake are depicted as boxplots in Fig. 3 and Supplementary Fig. S5. Each participant's concentrations are shown as representative examples for GHB, GHB-glycine and GHB-carnitine, 3,4-DHB, and GA (Supplementary Fig. S6). Significant differences between placebo and GHB treatment were observed for all analytes except SA and succinylcarnitine at the earliest collection time. Eleven hours post GHB administration, GHB, GHB-amino acids, 3,4-DHB, and GA still had significantly higher concentrations than placebo. 28 h after intake, only GHB-glycine allowed statistical discrimination. GHB-pentose and feature U4 showed some potential to distinguish exogenous from endogenous GHB.

Based on placebo concentrations, cut-off values were selected and tested for their sensitivity to detect GHB intake at the different time points post-administration. Results for specificity, sensitivity, and PPV are summarized in Table 2. Using the selected cut-offs, GHB-glycine performed better compared to GHB (sensitivity 0.4 vs. 0.1) or other biomarkers (e.g., 3,4-DHB sensitivity 0.1) up to at least 11 h. After 28 h, even GHB-glycine allowed GHB detection/discrimination from endogenous levels in only 9% of the cases, though.

**Table 2:** Proposed discrimination criteria (cut-off or elevation threshold) for GHB and its tested biomarkers, number of respective true positives (TP), false positives (FP), true negatives (TN), false negatives (FN), and their calculated specificity (spec, (TN/TN + FP)), sensitivity (sens, (TP/(TP + FN)), and the positive predictive value (PPV = TP/(TP + FP)) from study cohort II at 4.5 h, 11 h and 28 h following placebo or GHB intake.

Analyte	Discrimination criteria	TN	FP	TP	FN	Spec	Sens	PPV	TN	FP	TP	FN	Spec	Sens	PPV	TN	FP	TP	FN	Spec	Sens	PPV
		4.5 h							11 h							28 h						
GHB	Strategy a: Cut-off (6 µg/mL)	40	0	40	0	1.0	1.0	1.0	35	0	5	32	1.0	0.1	1.0	34	0	2	36	1.0	0.1	1.0
	Strategy a: Cut-off (10 µg/mL)	40	0	39	1	1.0	1.0	1.0	35	0	3	34	1.0	0.1	1.0	34	0	0	35	1.0	0.0	
	Strategy c: Ratio urine1/urine2 (> 5)	35	4	39	0	0.9	1.0	0.9	35	4	11	24	0.9	0.3	0.7	35	4	2	31	0.9	0.1	0.3
GHB-carnitine	Strategy a: Cut-off 1 (1 µg/mL)	40	0	34	6	1.0	0.9	1.0	34	1	0	37	1.0	0.0	0.0	34	0	2	36	1.0	0.1	1.0
	Strategy c: Ratio urine1/urine2 (> 5)	35	4	36	3	0.9	0.9	0.9	35	4	3	32	0.9	0.1	0.4	35	4	2	31	0.9	0.1	0.3
GHB-glycine	Strategy a: Cut-off (1 µg/mL)	40	0	40	0	1.0	1.0	1.0	35	0	13	24	1.0	0.4	1.0	33	1	3	31	1.0	0.1	0.8
	Strategy b: MR (> 2.5)	27	9	0	40	0.8	0.0	0.0	31	2	10	24	0.9	0.3	0.8	26	3	11	22	0.9	0.3	0.8
	Strategy c: Ratio urine1/urine2 (> 5)	33	6	39	0	0.8	1.0	0.9	33	6	19	16	0.8	0.5	0.8	33	6	18	15	0.8	0.5	0.8
GHB-glutamate	Strategy a: Cut-off (1.5 µg/mL)	40	0	35	5	1.0	0.9	1.0	35	0	0	37	1.0	0.0		34	0	0	35	1.0	0.0	
	Strategy c: Ratio urine1/urine2 (> 5)	32	7	37	2	0.8	0.9	0.8	32	7	10	25	0.8	0.3	0.6	32	7	9	24	0.8	0.3	0.6
GHB-phenylalanine	Strategy a: Cut-off (0.0025 µg/mL)	40	0	40	0	1.0	1.0	1.0	35	0	7	30	1.0	0.2	1.0	34	0	0	35	1.0	0.0	
	Strategy c: Ratio urine1/urine2 (> 5)	30	9	39	0	0.8	1.0	0.8	30	9	19	16	0.8	0.5	0.7	30	9	15	18	0.8	0.5	0.6
GHB-taurine	Strategy a: Cut-off (0.05 µg/mL)	40	0	37	3	1.0	0.9	1.0	35	0	4	33	1.0	0.1	1.0	34	0	0	35	1.0	0.0	
	Strategy c: Ratio urine1/urine2 (> 5)	38	1	39	0	1.0	1.0	1.0	38	1	17	18	1.0	0.5	0.9	38	1	14	19	1.0	0.4	0.9
GHB-pentose	Strategy c: Ratio urine1/urine2 (> 5)	36	3	39	0	0.9	1.0	0.9	36	3	31	4	0.9	0.9	0.9	36	3	31	2	0.9	0.9	0.9
GHB-glucuronide	Strategy a: Cut-off (3 µg/mL)	40	0	0	40	1.0	0.0		35	0	0	37	1.0	0.0		34	0	0	35	1.0	0.0	
	Strategy c: Ratio urine1/urine2 (> 5)	39	0	3	36	1.0	0.1	1.0	39	0	3	32	1.0	0.1	1.0	39	0	1	32	1.0	0.0	1.0
GHB-sulfate	Strategy c: Ratio urine1/urine2 (> 5)	39	0	5	34	1.0	0.1	1.0	39	0	5	30	1.0	0.1	1.0	39	0	7	26	1.0	0.2	1.0
U3	Strategy c: Ratio urine1/urine2 (> 5)	38	1	9	29	1.0	0.2	0.9	38	1	3	32	1.0	0.1	0.8	38	1	3	30	1.0	0.1	0.8
U4	Strategy c: Ratio urine1/urine2 (> 5)	39	0	24	15	1.0	0.6	1.0	39	0	12	23	1.0	0.3	1.0	39	0	9	24	1.0	0.3	1.0
U16	Strategy c: Ratio urine1/urine2 (> 5)	39	0	22	17	1.0	0.6	1.0	39	0	9	25	1.0	0.3	1.0	39	0	5	28	1.0	0.2	1.0
2,4-DHB	Strategy a: Cut-off 1 (25 µg/mL)	40	0	12	28	1.0	0.3	1.0	35	0	2	35	1.0	0.1	1.0	34	0	1	34	1.0	0.0	1.0
	Strategy a: Cut-off (36 µg/mL)	40	0	7	33	1.0	0.2	1.0	34	0	0	37	1.0	0.0		34	0	0	35	1.0	0.0	
	Strategy c: Ratio urine1/urine2 (> 5)	38	1	19	20	1.0	0.5	1.0	38	1	7	28	1.0	0.2	0.9	38	1	5	28	1.0	0.2	0.8
3,4-DHB	Strategy a: Cut-off (40 µg/mL)	39	1	32	8	1.0	0.8	1.0	35	0	5	32	1.0	0.1	1.0	34	0	1	34	1.0	0.0	1.0
	Strategy a: Cut-off (120 µg/mL)	40	0	32	8	1.0	0.8	1.0	35	0	5	32	1.0	0.1	1.0	34	0	1	34	1.0	0.0	1.0
	Strategy c: Ratio urine1/urine2 (> 5)	39	0	33	6	1.0	0.8	1.0	39	0	8	27	1.0	0.2	1.0	39	0	4	29	1.0	0.1	1.0
GA	Strategy a: Cut-off (160 µg/mL)	40	0	17	23	1.0	0.4	1.0	35	0	0	37	1.0	0.0		34	0	0	35	1.0	0.0	
	Strategy a: Cut-off (400 µg/mL)	40	0	17	23	1.0	0.4	1.0	35	0	0	37	1.0	0.0		34	0	0	35	1.0	0.0	
	Strategy c: Ratio urine1/urine2 (> 5)	39	0	24	15	1.0	0.6	1.0	39	0	3	32	1.0	0.1	1.0	39	0	1	32	1.0	0.0	1.0
GA-taurine	Strategy c: Ratio urine1/urine2 (> 5)	39	0	21	18	1.0	0.5	1.0	39	0	4	31	1.0	0.1	1.0	39	0	2	30	1.0	0.1	1.0
SA	Strategy a: Cut-off (30 µg/mL)	40	0	3	37	1.0	0.1	1.0	35	0	0	37	1.0	0.0		33	1	0	35	1.0	0.0	0.0
	Strategy a: Cut-off (60 µg/mL)	40	0	0	40	1.0	0.0		35	0	0	37	1.0	0.0		34	0	0	35	1.0	0.0	
	Strategy c: Ratio urine1/urine2 (> 5)	38	1	1	38	1.0	0.0	0.5	38	1	2	33	1.0	0.1	0.7	38	1	4	29	1.0	0.1	0.8
Succinylcarnitine	Strategy a: Cut-off (25 µg/mL)	40	0	0	40	1.0	0.0		35	0	0	37	1.0	0.0		34	0	2	36	1.0	0.1	1.0
	Strategy c: Ratio urine1/urine2 (> 5)	39	0	1	38	1.0	0.0	1.0	39	0	2	33	1.0	0.1	1.0	39	0	7	26	1.0	0.2	1.0

### Differentiation based on metabolite ratios (strategy b)

**Figure 4:**
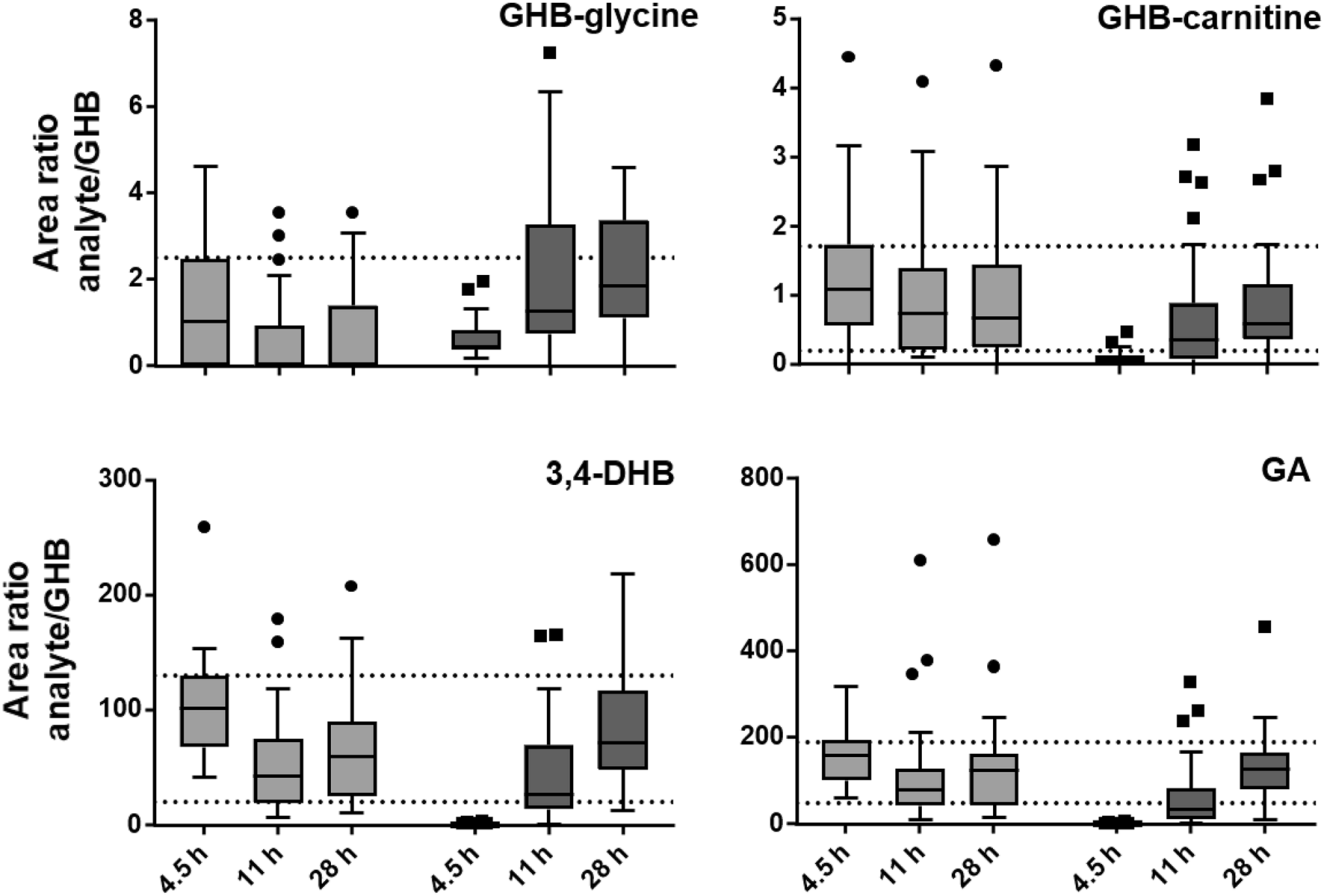
Metabolite ratios (analyte/GHB concentration) for placebo (light grey) and GHB treatment (dark grey) calculated from urine samples in study II at 4.5, 11, and 28 h after intake. Dotted lines represent the highest and lowest interquartile range of placebo treatment over all collection time points.

Metabolite ratios (metabolite/GHB, MR) increased from 4.5 to 28 h after GHB, but not placebo treatment as shown representatively for GHB-glycine, GHB-carnitine, 3,4-DHB, and GA in Fig. 4. The highest GHB concentrations were observed at 4.5 h after GHB administration and led to significantly decreased MRs compared to placebo. Only GHB-glycine interquartile ranges (IQR) exceeded those observed under placebo conditions. Taking the highest IQR MR of placebo treatment (2.5) as a tentative cut-off resulted in a lower sensitivity (0.3) and specificity (0.9) compared to strategy a) at 11 h. Sensitivity (0.3) with similar specificity was improved after 28 h.

### Differentiation based on elevation thresholds between two urine samples (strategy c)

Additionally, we evaluated the usefulness of elevated analyte levels (> 5) between two (subject- and time-matched) urine samples. Overall, better sensitivities were observed compared to a general cut-off application (strategy a; Table 2). After 28 h, about 50% or even 90% of the GHB-positive cases would be classified correctly by GHB-glycine or GHB-pentose, respectively. However, slightly lower specificity was detected for GHB-glycine because, in some urine pairs, one sample was negative for the corresponding biomarker.

## Discussion

The current interpretation of GHB urine concentrations still needs improvement, and several studies were performed to find new biomarkers^8–12,15–19,22,23^. We recently proposed new urinary GHB conjugates with different amino acids or carnitine (Fig. 1)^17,18^, but quantitative data over longer time intervals after GHB intake are missing. The current study is the first to provide quantitative values for GHB, its conjugates, and organic acids following controlled GHB administration to humans.

### Study cohorts, analytics, and detected concentrations

Urine samples from two study cohorts (79 participants) were (re)analyzed. While study cohort II consisted of a recent clinical trial (approximately 6 months storage at − 80 °C), study cohort I was stored for ca. 8 years (− 80 °C) before quantification in the current study. Long-term stability during method validation was tested for up to three months at − 20 °C. A trend towards declining concentrations of amino acid conjugates was observed, but only GHB-carnitine significantly decreased^21^. Accordingly, the mean and range of GHB amino acid conjugates in urine samples collected 4.5 h after GHB intake in study cohort I were slightly lower than in cohort II. Overall, these findings prove sufficient stability of GHB amino acid conjugates, at least when stored at − 80 °C. Surprisingly, we found significantly higher concentrations for GHB-carnitine in cohort I vs. II (Table 1). However, GHB-carnitine has proven unstable also in extracted samples. The substance is highly hygroscopic and challenging to handle under standard laboratory conditions. To avoid interpretative issues due to sample storage, we focused the primary data evaluation on study cohort II, given the long, matched sample collection.

The naturally occurring 13C isotope is usually about 1.1% of all carbon atoms producing a much lower MS signal than 12C, which has been shown to increase the dynamic range^24,25^. We could show during method validation that the GHB concentrations calculated via the ^13^C-isotope matched well with results of a previous GHB method^21^. The main focus of the present study was on new GHB metabolites, particularly on the differentiation between endogenous levels and exogenous GHB intake. We, therefore, considered the ^13^C-results as approximate values for GHB concentrations in samples exceeding the calibration range as sufficient and omitted further dilution steps.

Whether quantitative urinary concentrations should be adjusted to creatinine levels is critically discussed^26^. In spot-urine samples, creatinine adjustment is often recommended to account for individual dilution status^27^. In clinical and forensic toxicology, urinary creatinine is typically determined as a validity parameter^28,29^, but interpretation of urinary drug concentrations commonly does not consider creatinine levels. We observed a strong correlation between creatinine-adjusted and unadjusted concentrations, except for DHB isomers. Accordingly, we focused on results and discussion on unadjusted concentrations.

We detected GHB conjugates in all conditions and collection time points, although not in all individual urine samples (Table 1). Age, sex, or depressive disorder did not influence concentrations. Only fatty acid esters remained undetectable in all urine samples, most likely because of their high lipophilicity and the resulting poor renal excretion.

To differentiate endogenous levels from residues of exogenous application, knowledge of endogenous levels and their possible intra-/interday fluctuations is required. We found significant fluctuations only for succinylcarnitine, while DHB isomers and GHB glycine showed a slight, non-significant trend for higher early morning levels. Overall, GHB organic acid concentrations in placebo samples were in similar ranges as previously published by Kim et al.^13^ and generally slightly lower compared to Jarsiah et al.^12^ For new GHB conjugates, we provide first data. Still, more samples without the application of GHB need to be analyzed to determine endogenous levels reliably. Future studies might also aim for even lower LOQs for GHB-phenylalanine and GHB-taurine.

Following GHB treatment, we found the highest concentrations at the earliest sampling time. For organic acids, our results from this larger cohort align with previous findings by Kueting et al. following GHB intake in five narcoleptic patients (22–34 mg/kg body weight)^16^. However, compared to a limited number of authentic cases (Table 1, ForTox pos 1^21^), concentrations determined after controlled administrations (4.5 h) were lower for all compounds except for GHB-carnitine. Jarsiah et al. determined similar concentrations for DHB isomers and GA in forensic GHB cases (Table 1, ForTox pos 2^15^). Possible explanations include earlier urine collection or higher GHB doses consumed/administered. Sampling time in relation to drug intake and ingested doses are most often unknown in forensic cases. Sample storage and analyte stability might be critical as well. Recent long-term stability experiments over three months indicated significant increases in 2,4- and 3,4-DHB levels in GHB-positive but not in GHB-negative cases. However, this effect was not shown for GA or GHB conjugates^21^.

Concentrations of GHB metabolites, with the exceptions of GHB-carnitine, SA, and succinylcarnitine, showed a high correlation to those of GHB, particularly at the highest concentrations obtained through exogenous application. We observed a weaker correlation at later time points (28 h), where concentrations have a high probability of being of endogenous origin, especially for organic acids (Fig. 2). For 3,4-DHB, slopes between correlations of exogenous and endogenous origin seem to differ. Conjugate or organic acid concentrations various hours after GHB intake were not dependent on the initial GHB concentration. However, unfortunately, only three time points were available which did not allow calculation of elimination rates and underlying kinetics. Secondly, the first urine sample was collected at 4.5 h, which does not necessarily reflect the maximum concentration reached in urine.

Therefore, based on the available controlled data it cannot be fully excluded that higher doses of GHB will (not) result in higher concentrations of GHB conjugates, which may also be associated with longer detectability.

### Differentiation based on concentrations/ cut-off values (strategy a)

Mean concentrations of GHB were below the recommended GHB cut-offs of 10 or 6 µg/mL already 11 h post intake, which is in line with known detection windows of GHB^4^. Nevertheless, concentrations were still statistically significantly higher than after placebo. Initial explorative biomarker search^17,18^ raised hope that GHB amino acid conjugates do not occur endogenously, making them ideal candidates as exogenous GHB markers. However, the present study's more sensitive analytical detection methods also revealed low endogenous levels of these conjugates. GHB-pentose was the only analyte barely present in placebo urine samples (< 10%) but still detectable after 28 h (Supplementary Fig. S6). Unfortunately, no reference material is available and without method validation and quantification for this parameter, conclusions remain limited. The same applies for the still-unknown compound U4. Being clearly related to GHB administration, structure elucidation is essential for further studies^17^.

Based on the highest endogenous concentration per analyte detected in urine samples from placebo treatment (equal to 100% specificity), we have chosen cut-off values for GHB metabolites and tested them for their sensitivity to detect GHB intake at different time points. Additionally, if concentrations in authentic samples (Table 1) exceeded those in the controlled study cohorts, a second, higher cutoff, equal or very similar to a previous publication was evaluated^15^. GHB-glycine (tentative cut-off 1 µg/mL) stands out as the most promising biomarker that can prolong the detection window of GHB to about 28 h, but still not with the desired sensitivity of ideally close to one. Kueting et al. described elevated urinary creatinine-adjusted concentrations of 2,4-DHB, 3,4-DHB, and GA following GHB intake for up to 22 h compared to a patient-matched initial endogenous concentration before GHB intake^16^. These findings could not be confirmed in our data set. Only 3,4-DHB allowed correct identification of GHB intake for approximately 11 h, but still with low sensitivity (14%). Interestingly, the same urine samples (H19, H20, H21, P21, and P25) had higher concentrations for GHB, GHB-glycine, GHB-pentose, and 3,4-DHB compared to the other participants, especially at 11 h and sometimes after 28 h (Supplementary Fig. S6). So far, this finding could not be explained, but was shown to be independent of age, sex, co-medication or urinary creatinine.

### Differentiation based on metabolite ratios (strategy b)

Administration of GHB results in multiple times higher GHB urine concentrations compared to endogenous levels. Consequently, calculating MRs of GHB metabolites over GHB concentrations will initially yield extremely low MRs compared to placebo, increasing to or exceeding endogenous ratios over time (Fig. 4). If GHB metabolites are eliminated slower than GHB, the corresponding MRs at later time points should exceed those from cases without GHB intake. From all MRs evaluated, only GHB-glycine followed that expected trend, while median values still fell within the placebo limits. Selecting the highest IQR MR of placebo treatment as a tentative limit resulted in slightly superior, but still too low sensitivity for correct detection of GHB intake in 28 h urine samples compared to single concentration cut-off.

### Differentiation based on elevation thresholds between two urine samples (strategy c)

As a third discrimination approach, we tested a ratio determination between two urine specimens from the same individual, accounting for an individual’s endogenous level. We used time-matched placebo samples for a preliminary evaluation to avoid intra-day analyte variation. The analytical method was not sensitive enough to obtain quantitative values in all placebo samples for GHB conjugates. Ratio formation based on calculated concentrations was therefore not possible in sufficient cases. Instead, we used peak area ratios (analyte/internal standard (IS)), with the additional advantage that no reference material is needed. Such standards were partly synthesized in-house and are not yet commercially available^30^. Abundance differences in placebo urine samples (4.5 h vs. 28 h) were used as a cohort without exogenous GHB and showed maximum elevations of 5 (exception GHB, GHB-glutamate, GHB-phenylalanine, SA). Five was therefore selected as a potential elevation threshold. Compounds not detectable in individual samples were given a fictive peak area/IS ratio of 0.00001 (minimum factor 100 lower than lowest genuine sample values) to circumvent division through zero errors. This approach yielded much higher sensitivity, also 28 h after GHB intake. Poorer specificity values were observed caused by (placebo) urine pairs where analytes were undetectable in one of the samples. Under these circumstances, already very low levels in the first urine samples would lead to a theoretically infinite elevation. Taking GHB-glycine as an example, removing the six pairs with undetectable GHB-glycine in one of the samples increased specificity again from 0.85 to 1. Our preliminary data suggest that comparison to a second urine sample might be the best strategy to improve GHB detection. Still, more data, confirming proposed elevation thresholds, and evaluating results from duplicate routine case samples are necessary. Therefore, we recommend collecting urine samples 24 h (time-matched) after the initial sample.

### Limitations

Our study had some limitations as the reanalyzed samples came from studies not initially designed for our research question. In addition, a therapeutic (narcolepsy) GHB dose was administered, while higher doses are commonly used in DFSA cases. Only three collection time points up to 28 h were available, not allowing proper pharmacokinetic analysis (e.g., elimination rate).

## Conclusions

We provide the first comprehensive data on various GHB biomarkers following controlled administration of GHB. GHB-AA conjugates and 3,4-dihydroxybutyric acid proved suitable as additional GHB detection markers. Consequently, current GHB analysis should be extended from GHB only to several other biomarkers. Only GHB-glycine showed prolonged detection over GHB, though. Best sensitivities were obtained when a urine sample was compared to a second time- and subject-matched urine samples (strategy c). Still, collecting a second urine sample of DFSA victims might be critical, and (more) routine samples need to be analyzed with this approach before final evaluation.

## Materials and methods

### Study designs

We used urine samples from two randomized, balanced, double-blinded, placebo-controlled crossover studies performed in Zurich, Switzerland. They were initially designed to characterize the sleep-promoting effects of GHB in healthy participants (study I) and memory-enhancing effects in healthy participants and patients with major depressive disorders (study II). Approval was granted by the Cantonal Ethics Committee of Zurich and the Swissmedic. The studies were registered at ClinicalTrials.gov (NCT02342366 and NCT04082806, respectively) and all the study protocols and methods were in accordance with relevant guidelines and the declaration of Helsinki. GHB (50 mg/kg bodyweight Xyrem^®^) or placebo were administered dissolved in 2 dL of orange juice. Between sessions (placebo and GHB), a washout phase of seven days was maintained. All participants were instructed about potential risks and provided written informed consent, according to the declaration of Helsinki.

### Study cohort I

The study design is described in detail elsewhere^31^. Briefly, in the main study (n = 20 healthy males, mean age 25.8 ± 2.5, S01–S20), early morning urine samples were collected at 7:00 a.m. (4.5 h) following GHB/placebo administration at 2:30 a.m. of the experimental night. In an initial pilot study, GHB/placebo were administered the same way, but at 11 p.m. of the experimental night. Early morning urine (n = 20, SP01–SP20) was collected at 7:00 a.m. (8 h). All samples were stored at − 80 °C for approximately eight years and underwent a maximum of two freeze–thaw cycles. These urine samples were already used for former untargeted metabolome investigations^17,18^.

### Study cohort II

The study cohort II was obtained from a recent clinical drug administration study in healthy volunteers (H, n = 21, 6 males, 15 females, mean age 27 ± 6.6) and patients with major depressive disorder (P, n = 19, 6 males, 13 females, mean age 27 ± 6.9). Urine samples collected from the experimental night in the early morning (4.5 h ± 1 h), in the afternoon (11 h ± 1 h), and the following morning (28 h ± 1 h), were included in the current GHB quantification experiments. Samples were stored at − 80 °C for approximately 6 months until analysis and underwent a maximum of two freeze–thaw cycles. Further study details addressing the original study objectives will be published elsewhere.

### LC–MS/MS analysis for GHB and GHB metabolites

Chemicals and solvents used and a detailed description of the validated LC–MS/MS method are provided in reference^21^. Authentic urine samples (100 µL) were spiked with 10 µL IS solution (GHB-d_6_ 5 µg/mL; butyrylcarnitine-d3 5 µg/mL). All samples were diluted with 500 µL acetonitrile, centrifuged (14,000 rpm, 15 min) and the supernatant transferred to autosampler vials. Analysis was performed on a Shimadzu LC-40Dx3 LC system (Shimadzu, Duisburg/Germany) coupled to a Sciex 5500 QTtrap linear ion trap quadrupole MS (Sciex, Darmstadt/Germany) controlled by Analyst software (version 1.7.2). Briefly, chromatographic separation was performed on a SeQuant ZIC-HILIC column (Merck, Darmstadt/Germany) following gradient elution. The flow rate was 0.35 mL/min. The MS was operated in advanced, scheduled multiple reaction monitoring mode using one to three transitions per analyte, including ^13^C-isotopes for GHB and GHB-carnitine. Calibrators were prepared in synthetic urine.

### Creatinine determination

Creatinine was determined by the Jaffe reaction on an Indiko Plus device (Thermo Scientific, Braunschweig/Germany).

### Data evaluation

MultiQuant software (3.0.3, Sciex) was used for peak integration and quantification. Statistical analysis was performed in GraphPad Prism 7.0 (GraphPad Software, San Diego/CA/USA). Group comparisons (placebo vs. GHB; different time points post intake) were performed in study cohorts I and II by one-tailed Mann–Whitney or Kruskal–Wallis test followed by Dunn’s multiple corrections test (p < 0.05). The influence of sex and disease was evaluated using two-way ANOVA with Sidak’s multiple comparison test. We tested correlation including age with spearman correlation analysis.

### Strategies for expanding the detection window

Different strategies were evaluated for their ability to discriminate between GHB treatment and endogenous levels in study cohort II: (a) cut-off concentrations of the new GHB metabolites, (b) metabolic ratios (MR, metabolite concentration/GHB concentration), and (c) elevated peak area ratios (analyte/IS) between two urine samples. Urine samples following GHB treatment were taken as urine 1, and time-matched placebo samples as urine 2. A data set without exogenous GHB was formed through best time-matched placebo samples at 4.5 h (urine 1) or 28 h (urine 2). To assess the quality of the strategies, the number of true positives (TP), false positives (FP), true negatives (TN), false negatives (FN), resulting sensitivity (TP/(TP + FN), specificity (TN/TN + FP), and the positive predictive value (PPV = TP/(TP + FP)) were calculated.

## Supplementary Information

Supplementary Information.

## Data Availability

The datasets generated during and/or analyzed during the current study are available from the corresponding author on reasonable request.
