# Supplementary material for: Urinary concentrations of GHB and its novel amino acid and carnitine conjugates following controlled GHB administration to humans
Source: Sci Rep. 2023 Jun 2;13:8983. doi: 10.1038/s41598-023-36213-1 (PMC10238486; doi:10.1038/s41598-023-36213-1)
Supplement: Supplementary file 1 — Supplementary Information. [file 41598_2023_36213_MOESM1_ESM.pdf]

Supplementary Information

Prolonged GHB detection? - Urinary concentrations of GHB and its novel amino acid and carnitine conjugates following controlled GHB administration to humans

Andrea E. Steuer[1], Francesco Bavato[2], Laura K. Schnider[2], Dario A. Dornbierer[1,2], Oliver G. Bosch[2], Boris B. Quednow[2,3], Erich Seifritz[2,3], Christian Steuer[4], Thomas Kraemer[1]

[1]*Department of Forensic Pharmacology & Toxicology, Zurich Institute of Forensic Medicine, University of Zurich, 8057 Zurich, Switzerland*

[2]*Department of Psychiatry, Psychotherapy and Psychosomatics, Psychiatric University Hospital Zurich, University of Zurich, 8032 Zurich, Switzerland*

[3]*Neuroscience Center Zurich, University of Zurich and Swiss Federal Institute of Technology Zurich, 8057 Zurich, Switzerland*

[4]*Institute of Pharmaceutical Sciences, Swiss Federal Institute of Technology Zurich, 8093 Zurich, Switzerland*

Corresponding author

Andrea Steuer

Department of Forensic Pharmacology and Toxicology, Institute of Forensic Medicine,

University of Zurich, Winterthurerstrasse 190/52, 8057 Zurich, Switzerland

+41 446355679

andrea.steuer@irm.uzh.ch

Figure S1. Correlation of GHB concentration determined via calibration using the ^{12}C-isotope (x-axis) vs. its ^{13}C-isotope (y-axis). The solid line represents perfect correlation, the dotted line indicates the concentration of the highest calibrator (82.5 µg/mL).

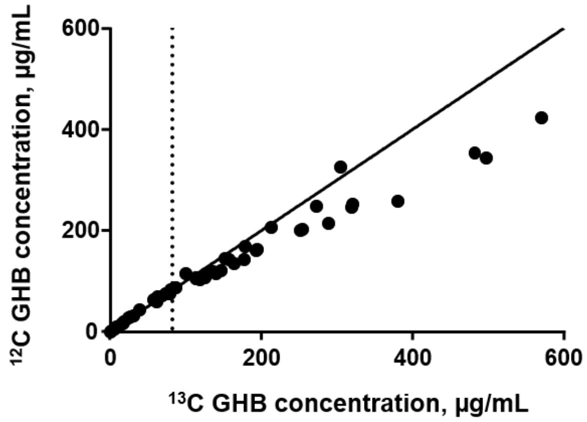

Figure S2. Box plots of urinary creatinine concentrations of studies I and II according to treatment condition (A), gender (B) and time after Placebo/GHB intake (C). Placebo treatment is given in light grey, GHB intake in dark grey. Statistical comparison was performed using Kruskal-Wallis test, followed by Dunn's multiple comparisons post-hoc test or two-way ANOVA with Sidak's multiple comparison test ($p < 0.05$): ** $p < 0.01$; *** $p < 0.001$; **** $p < 0.0001$.

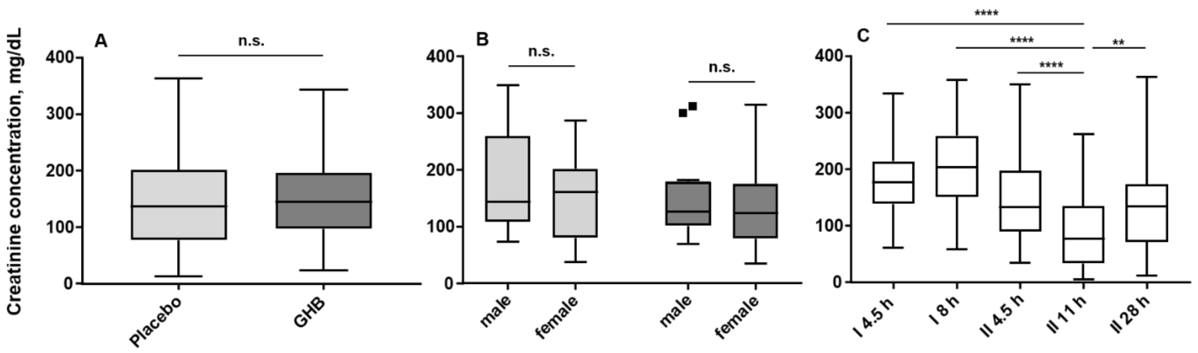

Figure S3. Correlation analysis (spearman, r) between unadjusted (x-axis) and creatinine-adjusted (y-axis) urinary concentrations. Solid lines represent results for linear correlation.

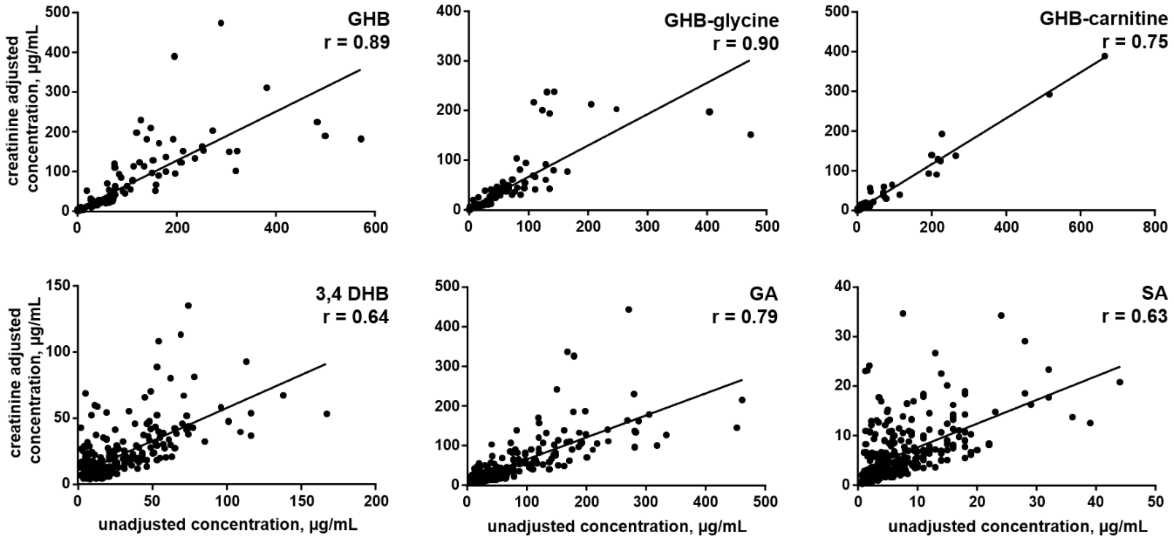

Figure S4. Box plots of urine concentrations divided by sex for placebo (light grey) and GHB treatment (dark grey) collected in study II at 4.5 (upper pane) and 28 h (lower pane) after intake. Statistical comparison was performed using a two-way ANOVA with Sidak's multiple comparison test ($p < 0.05$): * $p < 0.05$; ** $p < 0.01$.

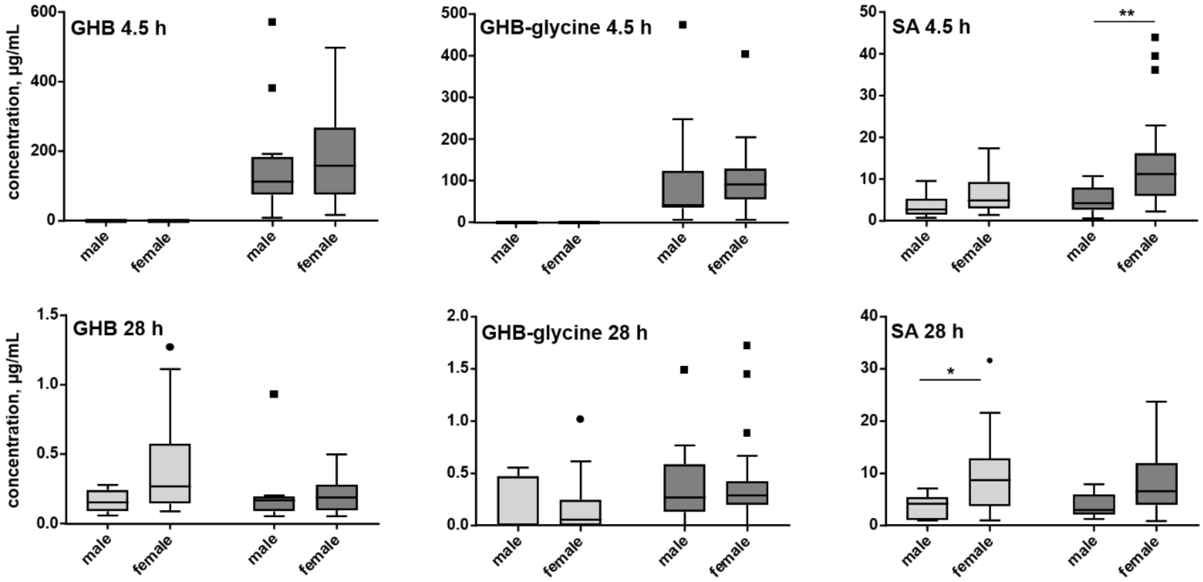

Figure S5. Box plots of urine concentrations (upper pane) or area ratios of analyte peak area over peak area of the respective internal standards (middle and lower pane) for placebo (light grey) and GHB treatment (dark grey) collected in study II at 4.5, 11, and 28 h after intake. Statistical comparison was performed using a one-tailed Mann-Whitney test ($p < 0.05$): * $p < 0.05$; ** $p < 0.01$; *** $p < 0.001$; **** $p < 0.0001$.

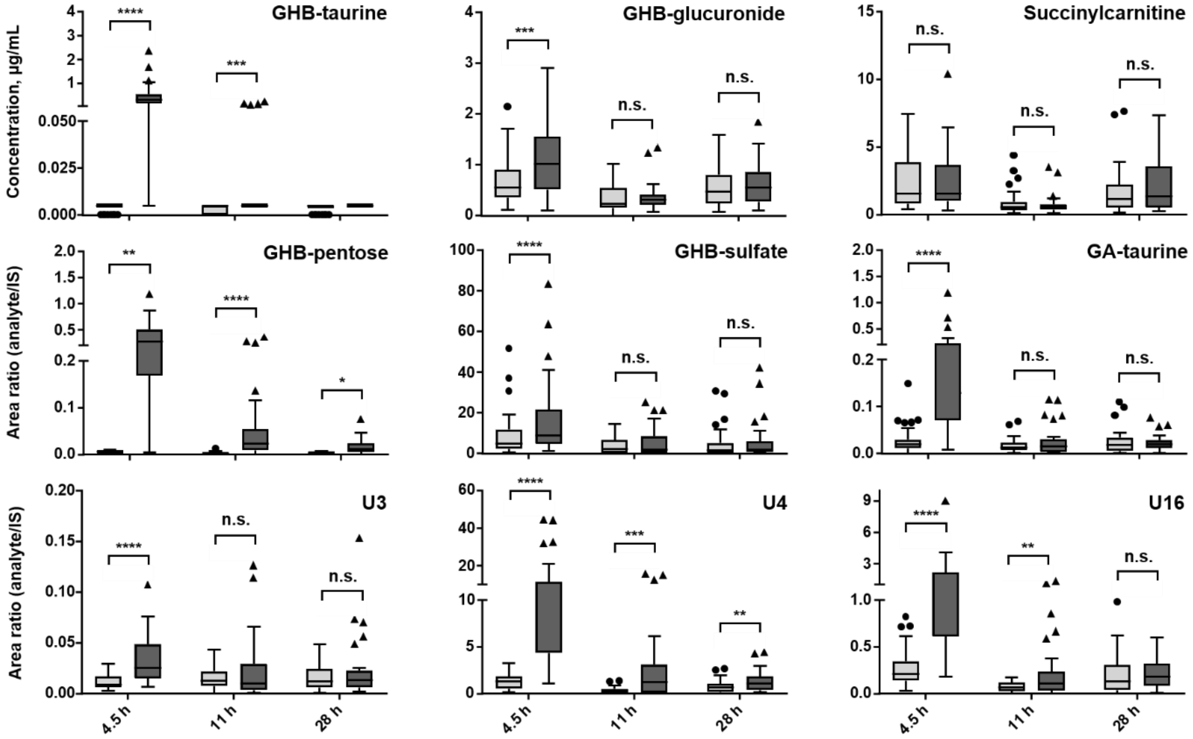

Figure S6. Time-dependent urinary levels of GHB and five selected metabolites in placebo (light grey, circles, dotted line) and GHB (blank triangles, solid lines) conditions at 4.5, 11, and 28 h following intake.

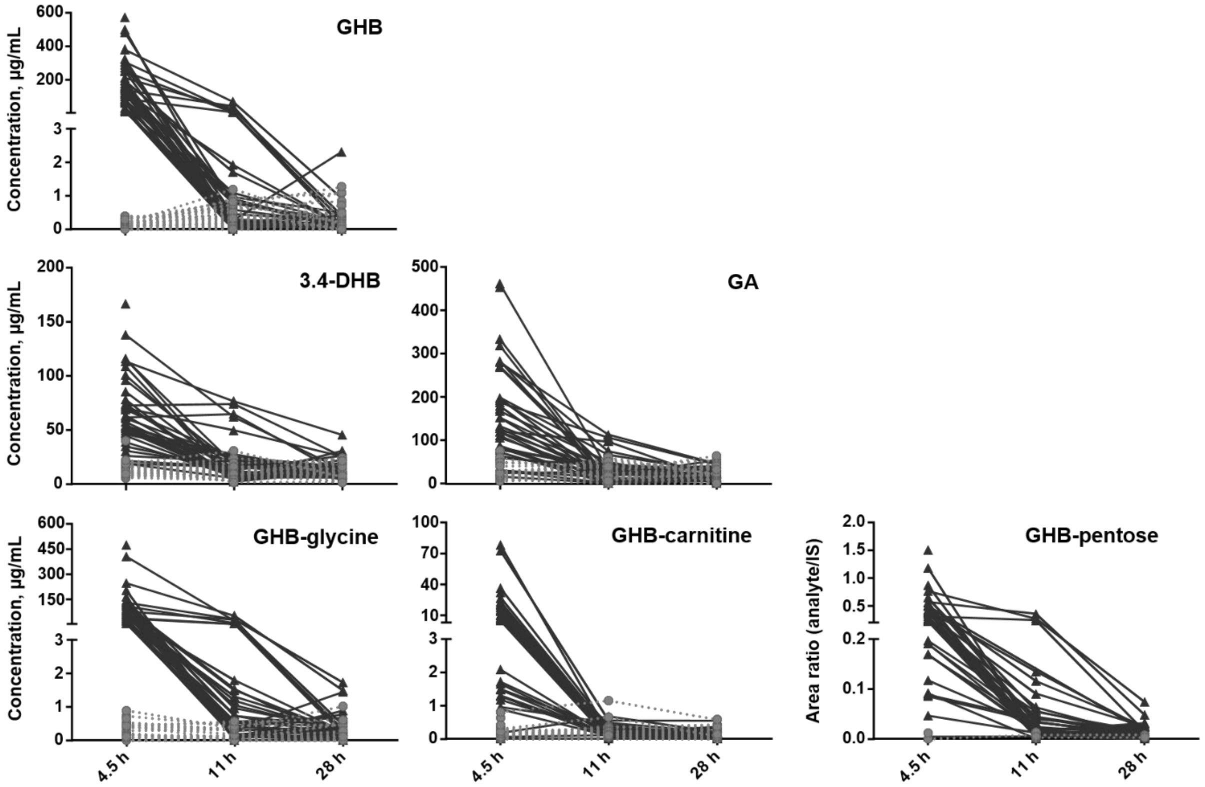

Table S1. Calculated urine concentrations (µg/mL) for GHB and its potential biomarker in each individual sample. For study I and pilot study I, urinary concentrations of GHB and potential biomarkers could be measured in 19 placebo and 19 GHB samples (19 pairs; study I, t = 4.5 h) and in 17 placebo and 18 GHB samples (15 pairs; study I pilot, t = 8 h), respectively. In study II, quantification was possible in 40 placebo and 40 GHB samples (40 pairs, t = 4.5h), 35 placebo and 37 GHB (35 pairs, t = 11 h) and 34 placebo and 35 GHB samples (33 pairs, t = 28 h), respectively. <LQ represents calculated concentration below the calibration curve, values exceeding the calibration curve are indicated in italic print. M, male, f, female.

Participant	Study	Sex	Sampling time, h	Creatinine, mg/dL	Creatinine, mg/dL	GHB		GHB carnitine		GHB glycine		GHB glutamate		*GHB* phenylalanine		*GHB* taurine		GHB glucuronide		2,4-DHB		3,4-DHB		GA		SA		Succinyl-carnitine	
				P	G	P	G	P	G	P	G	P	G	P	G	P	G	P	G	P	G	P	G	P	G	P	G	P	G
S01	1	m	4.5	126	143	0.11	*111*	0.30	*200*	0.07	57	0.13	1.9	*<LQ*	0.023	*<LQ*	0.15	0.46	1.1	8.0	20	15	48	43	146	1.9	2.8	3.4	2.2
S02	1	m	4.5	208	178	0.22	*91*	0.40	*224*	0.24	44	*n.d.*	2.3	*<LQ*	0.016	*<LQ*	0.12	1.5	3.2	9.7	22	17	49	68	148	4.5	10	1.6	2.0
S03	1	m	4.5	283	228	0.17	58	0.15	3.7	0.24	36	*n.d.*	2.1	*<LQ*	0.027	*<LQ*	0.09	1.8	2.2	11	18	18	41	60	104	5.6	6.5	7.6	5.3
S04	1	m	4.5	159	234	0.17	71	0.12	18	0.07	36	*n.d.*	2.2	*<LQ*	0.022	*<LQ*	0.10	0.48	1.3	13	23	21	44	77	138	1.8	9.6	1.8	2.8
S05	1	m	4.5	266	235	0.14	*158*	0.12	*213*	0.13	72	*n.d.*	4.4	*<LQ*	0.043	*<LQ*	0.31	1.8	1.5	8.5	*28*	16	*65*	46	211	6.4	18	12	2.6
S06	1	m	4.5	177	146	0.26	69	0.34	94	0.08	37	*n.d.*	1.2	*<LQ*	0.025	*<LQ*	0.08	0.75	0.69	9.6	18	17	41	56	121	2.0	4.2	1.3	2.1
S07	1	m	4.5	85	193	*<LQ*	65	0.07	*265*	*n.d.*	31	*<LQ*	1.5	*<LQ*	0.019	*<LQ*	0.09	0.33	1.4	2.4	19	3.7	36	16	187	*<LQ*	5.7	0.19	2.0
S08	1	m	4.5	334	171	0.41	*210*	0.42	*666*	0.26	94	*n.d.*	4.3	*<LQ*	0.037	*<LQ*	0.28	1.7	2.2	20	*31*	40	*66*	163	305	5.4	5.2	4.4	2.1
S09	1																												
S10	1	m	4.5	213	118	0.22	*134*	0.46	*228*	0.12	52	*n.d.*	2.1	*<LQ*	0.046	*<LQ*	0.12	0.79	0.72	20	19	27	49	62	163	3.1	4.3	2.6	1.1
S11	1	m	4.5	149	207	0.24	*197*	0.12	*193*	0.08	77	*n.d.*	2.5	*<LQ*	0.026	*<LQ*	0.30	0.68	1.8	11	*31*	17	*62*	52	218	4.9	15	2.1	2.6

S12	1	m	4.5	185	181	0.27	*163*	*n.d.*	5.7	*n.d.*	44	*n.d.*	1.9	*<LQ*	0.045	*<LQ*	0.15	0.39	0.99	6.0	27	14	*53*	66	190	11	32	0.82	2.4
S13	1	m	4.5	112	118	0.18	63	0.30	1.5	0.15	44	*<LQ*	1.2	*<LQ*	0.019	*<LQ*	0.10	0.42	0.87	9.1	15	16	44	42	97	1.9	5.4	1.4	2.1
S14	1	m	4.5	215	154	0.27	*150*	0.30	70	0.19	67	*n.d.*	2.8	*<LQ*	0.031	*<LQ*	0.20	1.1	1.8	16	26	22	*55*	65	167	9.7	28	0.74	2.1
S15	1	m	4.5	274	212	0.12	*95*	0.10	4.4	0.18	93	*n.d.*	5.9	*<LQ*	0.046	*<LQ*	0.43	1.6	2.5	12	*38*	16	*58*	65	237	10.0	14	26	13
S16	1	m	4.5	124	62	0.11	74	0.16	35	*n.d.*	16	*n.d.*	1.3	*<LQ*	0.018	*<LQ*	0.07	0.81	1.6	8.0	21	14	34	97	150	3.5	11	1.7	2.1
S17	1	m	4.5	61	177	0.21	*236*	*n.d.*	*517*	*<LQ*	142	*<LQ*	5.1	*<LQ*	0.062	*<LQ*	0.59	0.21	0.94	3.3	29	6.6	*73*	22	288	5.2	29	0.36	2.4
S18	1	m	4.5	125	197	0.06	73	0.10	36	*n.d.*	11	*n.d.*	1.2	*<LQ*	0.016	*<LQ*	0.05	0.57	1.2	11	19	9.1	34	42	149	4.7	16	0.80	1.3
S19	1	m	4.5	225	194	0.33	*107*	0.42	72	0.22	33	*n.d.*	2.2	*<LQ*	0.032	*<LQ*	0.12	1.2	2.4	16	24	22	43	80	176	5.1	12	5.7	5.2
S20	1	m	4.5	156	168	0.12	*207*	0.31	*217*	0.08	62	*n.d.*	4.1	*<LQ*	0.037	*<LQ*	0.24	0.72	1.6	11	27	15	*74*	63	236	4.5	15	2.1	2.6
SP01	1	m	8	90	59	0.14	1.4	0.07	0.06	0.02	0.79	0.07	0.14	*<LQ*	0.001	*n.d.*	*<LQ*	0.59	0.20	7.5	4.9	8.4	8.9	28	19	6.1	5.9	2.1	0.73
SP02	1	m	8	68	202	*<LQ*	4.1	0.20	0.64	0.06	8.7	0.06	*n.d.*	*<LQ*	0.003	*n.d.*	*<LQ*	0.44	1.4	2.9	17	6.0	40	22	75	0.75	5.8	1.0	6.7
SP03	1	m	8	349	343	0.10	12	0.19	10	0.12	16	*n.d.*	0.57	*n.d.*	0.003	*<LQ*	0.07	1.7	2.1	8.5	18	16	46	65	138	3.3	7.1	13	9.4
SP04	1	m	8		149		3.9		*n.d.*		1.2		*<LQ*		0.002		*<LQ*		0.70		8.2		15		51		3.8		0.80
SP05	1	m	8	138	179	0.06	76	0.17	71	0.12	31	*<LQ*	1.5	*<LQ*	0.017	*n.d.*	0.08	0.79	2.0	4.2	19	8.5	43	30	115	1.8	13	0.85	1.9
SP06	1	m	8	159	92	0.07	27	0.07	15	*<LQ*	7.7	*n.d.*	0.47	*<LQ*	0.010	*n.d.*	*<LQ*	0.72	0.82	8.2	11	13	30	52	76	1.7	1.4	4.5	1.3
SP07	1	m	8	260	274	0.31	28	0.30	5.4	0.14	25	*n.d.*	0.37	*<LQ*	0.008	*n.d.*	0.08	0.94	0.69	21	21	31	*55*	108	172	3.7	5.7	2.5	3.1
SP08	1	m	8	244	213	0.15	45	0.19	0.14	0.15	31	*n.d.*	1.0	*<LQ*	0.015	*n.d.*	0.12	1.3	0.88	8.8	22	15	*51*	87	142	6.0	11	2.7	7.6
SP09	1	m	8	357	245	0.33	40	0.39	21	0.35	18	*n.d.*	0.90	*<LQ*	0.014	*<LQ*	0.07	2.6	3.3	16	23	21	36	79	127	8.3	10	9.1	7.7
SP10	1	m	8	179	243	0.07	32	0.22	35	0.10	19	*n.d.*	1.1	*<LQ*	0.011	*n.d.*	0.08	0.71	0.93	6.3	19	12	41	43	108	2.4	7.1	1.1	3.2
SP11	1	m	8	166	158	0.13	36	0.16	18	0.10	16	*n.d.*	0.91	*<LQ*	0.012	*n.d.*	0.06	1.7	2.2	12	18	19	37	55	93	2.2	7.4	5.3	6.3
SP12	1	m	8	247	84	0.15	27	0.23	4.8	0.09	8.0	*n.d.*	0.50	*<LQ*	0.005	*<LQ*	*<LQ*	1.8	0.95	9.0	9.8	16	38	66	72	7.7	9.0	6.7	2.2
SP13	1	m	8	119		*<LQ*		0.08		*n.d.*		*n.d.*		*<LQ*		*n.d.*		0.26		4.1		8.5		39		2.3		2.0	

SP14	1	m	8	204	175	0.35	11	*n.d.*	6.3	*n.d.*	5.1	*n.d.*	0.26	*<LQ*	0.001	*n.d.*	*<LQ*	1.0	1.7	7.1	20	13	31	67	97	8.9	3.1	4.6	2.1
SP15	1	m	8	259	207	0.16	47	0.21	43	0.11	23	*n.d.*	0.80	*<LQ*	0.015	*<LQ*	0.13	2.9	3.1	8.2	18	12	41	39	117	5.0	16	2.3	1.1
SP16	1	m	8	173	188	0.09	57	0.20	17	0.07	23	*n.d.*	1.8	*<LQ*	0.017	*<LQ*	0.10	0.84	1.8	7.4	24	14	*57*	53	145	1.3	2.9	1.9	4.4
SP17	1	m	8	218	291	0.13	64	0.20	115	*n.d.*	32	*n.d.*	1.7	*n.d.*	0.006	*<LQ*	0.15	1.1	2.5	6.8	22	13	*59*	63	281	2.9	15	2.1	4.4
SP18	1	m	8		282		57		35		35		1.1		0.012		0.12		1.6		29		*53*		201		19		4.5
SP19	1	m	8	304		0.16		0.11		*n.d.*		*n.d.*		*n.d.*		*<LQ*		1.6		9.8		17		60		7.4		6.2	
SP20	1	m	8	258	146	0.14	21	0.09	8.1	*n.d.*	15	*n.d.*	0.70	*<LQ*	0.011	*<LQ*	*<LQ*	1.2	1.0	7.1	12	14	32	63	66	8.0	9.8	2.6	2.5
H01	2	f	4.5	74	163	0.06	16	0.20	2.1	*n.d.*	18	0.43	1.7	*<LQ*	0.004	*n.d.*	0.07	0.29	2.3	3.9	22	6.9	*52*	17	88	2.0	2.9	0.57	2.7
H01	2	f	11	40	24	0.17	*<LQ*	0.22	0.11	0.06	0.09	0.17	0.10	*<LQ*	*<LQ*	*<LQ*	*<LQ*	0.95	1.2	5.3	2.8	7.3	5.2	12	*<LQ*	5.3	3.1	1.2	3.5
H01	2	f	28	188		0.15		0.42		0.26		0.37		*<LQ*		*<LQ*		1.6		8.0		13		35		7.8		7.4	
H02	2	f	4.5	58	55	0.10	*127*	*n.d.*	*n.d.*	0.16	131	0.15	5.3	*n.d.*	0.013	*<LQ*	0.29	0.35	0.58	7.4	18	10	*74*	10	179	3.3	9.3	4.9	0.99
H02	2	f	11	27	7	0.35	0.29	0.05	*n.d.*	0.08	0.15	0.05	0.07	0.0011	*n.d.*	*<LQ*	*<LQ*	0.26	0.11	3.2	2.3	3.9	4.8	*<LQ*	*<LQ*	3.4	1.6	0.68	0.36
H02	2	f	28	73	75	0.13	0.16	*<LQ*	*n.d.*	*n.d.*	0.27	0.14	0.21	*<LQ*	0.0008	*<LQ*	*<LQ*	0.12	0.22	5.1	9.0	7.8	11	18	13	0.89	0.82	0.48	0.57
H03	2	m	4.5	271	158	0.16	*100*	0.41	0.32	0.21	41	0.21	0.76	*<LQ*	0.013	*n.d.*	0.32	0.33	0.10	8.7	16	15	30	52	173	2.5	0.66	1.2	0.31
H03	2	m	11																										
H03	2	m	28																										
H04	2	m	4.5	226	312	0.27	*571*	0.63	33	0.74	473	0.16	20	*n.d.*	*0.11*	*n.d.*	3.0	0.39	1.4	12	*43*	40	*167*	76	*453*	5.7	11	1.4	3.9
H04	2	m	11																										
H04	2	m	28																										
H06	2	m	4.5	73	106	0.08	*193*	*n.d.*	8.1	0.07	86	0.09	6.7	0.0005	0.053	*<LQ*	0.46	0.84	1.4	6.5	24	8.9	*71*	8.6	198	0.72	2.3	7.4	10
H06	2	m	11	48	33	0.50	0.24	0.09	0.13	0.10	0.46	0.06	0.08	*<LQ*	*<LQ*	*<LQ*	*<LQ*	0.38	0.27	6.5	3.8	17	12	11	7.1	3.0	0.72	0.94	0.66
H06	2	m	28	12	61	*<LQ*	0.05	0.06	*n.d.*	*<LQ*	0.19	*<LQ*	0.06	*n.d.*	*<LQ*	*n.d.*	*<LQ*	0.17	0.55	*<LQ*	4.0	3.0	6.4	*<LQ*	7.3	*<LQ*	<LQ	0.58	5.5

H07	2	f	4.5	74	89	0.07	*84*	0.06	11	*n.d.*	37	0.07	1.5	*<LQ*	0.007	*<LQ*	0.10	0.61	0.46	4.6	10	8.8	26	16	81	2.7	3.7	0.56	0.51
H07	2	f	11	79	111	0.16	0.78	0.08	0.18	*n.d.*	3.5	0.10	0.25	*<LQ*	0.003	*n.d.*	*<LQ*	0.43	0.08	4.9	8.7	8.7	21	14	35	7.2	2.0	0.38	0.17
H07	2	f	28	153	163	0.35	0.26	*n.d.*	0.15	0.14	0.29	0.11	0.18	*<LQ*	*<LQ*	*<LQ*	*<LQ*	0.84	0.36	11	8.3	16	15	24	36	12	11	0.94	0.71
H08	2	f	4.5	183	61	0.39	*289*	0.29	7.0	*n.d.*	123	0.11	9.2	*n.d.*	0.058	*<LQ*	0.49	0.65	0.83	8.7	19	17	*69*	61	271	9.7	6.1	1.5	1.2
H08	2	f	11	28	97	0.27	0.45	0.16	0.17	*n.d.*	0.74	0.05	0.23	*<LQ*	0.0009	*<LQ*	*<LQ*	0.31	0.31	3.7	12	6.6	25	12	32	2.9	8.6	2.2	0.69
H08	2	f	28	44	133	0.45	0.26	0.15	0.10	*n.d.*	0.10	0.06	0.12	*n.d.*	*<LQ*	*<LQ*	*<LQ*	0.56	0.45	4.7	10	7.5	16	21	34	2.6	6.9	1.5	1.1
H09	2	f	4.5	38	50	0.12	*195*	*n.d.*	0.06	0.07	109	*<LQ*	4.0	*<LQ*	0.042	*<LQ*	0.32	0.54	0.50	2.3	13	5.6	*54*	7.3	168	5.0	13	1.5	1.1
H09	2	f	11	76	109	0.90	1.7	0.17	0.12	0.16	1.5	0.06	0.23	0.0005	0.002	*<LQ*	*<LQ*	1.0	0.40	10	12	15	23	34	51	15	16	2.7	0.99
H09	2	f	28	84	23	1.1	0.13	0.06	*n.d.*	*n.d.*	*<LQ*	0.22	*<LQ*	0.0022	*<LQ*	*<LQ*	*<LQ*	0.19	0.25	7.7	1.9	14	4.2	45	*<LQ*	5.0	3.9	0.47	1.0
H10	2	f	4.5	282	100	0.10	*113*	0.11	1.3	0.47	95	0.07	6.3	*n.d.*	0.040	*<LQ*	0.32	1.1	1.6	7.6	20	15	*53*	15	120	4.6	16	4.6	1.6
H10	2	f	11	125	120	0.25	1.1	0.39	0.15	0.52	1.5	0.07	0.19	*<LQ*	0.001	*<LQ*	*<LQ*	1.0	0.63	11	11	24	25	26	30	18	15	1.7	1.0
H10	2	f	28	221	71	0.14	0.19	0.21	0.09	0.44	0.22	0.09	0.09	*n.d.*	*<LQ*	*<LQ*	*<LQ*	0.52	0.64	9.2	5.3	18	9.3	20	11	9.1	7.5	3.9	1.3
H11	2	f	4.5	154	118	0.10	*152*	0.17	21	0.28	73	0.09	2.6	*n.d.*	0.029	*<LQ*	0.28	0.42	1.6	6.9	20	13	*52*	27	107	2.5	16	0.52	0.87
H11	2	f	11	178	72	0.13	0.91	0.34	0.19	0.32	0.54	1.40	0.10	*<LQ*	*<LQ*	*<LQ*	*<LQ*	0.55	0.33	6.5	7.1	13	20	24	23	3.2	3.3	3.2	0.39
H11	2	f	28	170	210	0.15	0.10	0.43	0.37	*n.d.*	0.42	0.75	0.13	*<LQ*	*<LQ*	*<LQ*	*<LQ*	0.87	1.1	6.8	6.7	14	12	38	15	4.3	4.1	1.6	1.4
H12	2	f	4.5	169	211	0.16	*321*	0.12	14	*n.d.*	129	0.10	12	*<LQ*	0.098	*<LQ*	0.83	1.6	2.9	6.8	*39*	17	*101*	17	283	12	44	6.1	3.0
H12	2	f	11	110	22	0.39	0.21	0.26	0.12	*n.d.*	0.17	0.11	0.15	*<LQ*	*<LQ*	*<LQ*	*<LQ*	0.59	0.50	7.0	3.6	14	13	27	*<LQ*	11	7.6	0.95	1.2
H12	2	f	28	135	158	0.09	0.12	0.16	0.21	0.13	0.26	0.13	0.19	*n.d.*	*<LQ*	*<LQ*	*<LQ*	1.4	1.2	6.9	9.8	14	26	11	15	9.4	12	7.6	5.4
H13	2	f	4.5	67	68	0.06	75	0.06	1.3	*n.d.*	27	*n.d.*	2.5	*n.d.*	0.019	*<LQ*	0.16	0.23	0.34	3.9	12	7.6	45	5.4	73	3.5	9.0	5.2	1.2
H13	2	f	11	70	20	0.19	0.11	0.23	0.05	0.16	0.14	0.15	0.06	*<LQ*	*<LQ*	*<LQ*	*<LQ*	0.15	0.11	6.0	2.3	10	6.2	16	*<LQ*	5.5	3.5	0.56	0.65
H13	2	f	28	135	59	0.14	0.06	0.10	0.07	*n.d.*	0.14	*n.d.*	0.06	*n.d.*	*<LQ*	*<LQ*	*<LQ*	0.20	0.14	6.0	3.2	11	6.1	18	11	7.8	3.9	3.8	0.85
H14	2	f	4.5	274	179	0.29	*178*	0.05	1.7	*n.d.*	81	0.09	4.6	0.0006	0.023	*<LQ*	0.44	0.62	1.1	8.1	20	21	*61*	49	193	14	6.1	1.1	1.0

H14	2	f	11	57	193	0.65	0.27	0.08	0.21	*n.d.*	1.0	0.15	0.15	0.0007	0.0008	*n.d.*	*<LQ*	0.06	0.56	12	9.1	19	24	42	45	1.5	2.8	0.13	0.49
H14	2	f	28	257	96	0.46	0.10	*n.d.*	*n.d.*	*n.d.*	0.26	0.11	0.12	0.0007	*<LQ*	*<LQ*	*<LQ*	1.1	0.86	9.7	7.2	23	15	38	16	22	4.1	2.5	4.9
H15	2	f	4.5	89	134	*<LQ*	*273*	0.06	20	*n.d.*	67	0.07	1.8	*n.d.*	0.025	*n.d.*	0.21	0.26	0.46	3.5	23	8.1	*54*	15	153	3.1	4.6	0.46	0.58
H15	2	f	11	37	17	0.05	0.92	0.08	0.18	*n.d.*	1.2	*<LQ*	0.10	*n.d.*	0.0007	*n.d.*	*<LQ*	0.32	0.29	2.4	2.7	5.7	8.9	20	*<LQ*	4.5	2.2	0.50	0.56
H15	2	f	28	117	71	0.19	*<LQ*	0.10	*n.d.*	*n.d.*	0.21	0.10	0.07	*<LQ*	*<LQ*	*n.d.*	*<LQ*	0.31	0.49	5.0	4.3	11	7.5	34	13	11	6.1	0.55	3.4
H17	2	f	4.5	122	100	0.16	29	0.20	0.90	0.53	57	*n.d.*	2.1	*<LQ*	0.023	*<LQ*	0.08	0.86	0.84	8.0	23	11	48	15	31	9.4	7.8	4.6	6.1
H17	2	f	11	83	98	0.59	0.96	0.34	0.11	0.33	0.57	*n.d.*	0.07	*<LQ*	0.0006	*<LQ*	*<LQ*	0.23	0.39	5.0	14	8.7	21	9.8	13	12	28	0.55	0.97
H17	2	f	28		129		0.50		0.11		0.67		*n.d.*		*n.d.*		*<LQ*		0.87		12		16		6.3		14		7.4
H18	2	m	4.5	108	182	0.07	8.8	0.08	1.5	0.17	6.2	*n.d.*	0.29	*n.d.*	0.004	*<LQ*	*<LQ*	0.78	0.73	6.0	8.0	11	19	12	24	1.9	3.2	4.5	2.2
H18	2	m	11	87	153	0.16	0.59	0.14	0.29	0.12	0.28	0.08	0.07	*<LQ*	*<LQ*	*<LQ*	*<LQ*	0.16	0.22	4.0	8.2	6.6	16	14	28	2.7	7.1	0.37	0.55
H18	2	m	28	136	113	0.16	0.18	0.10	0.21	0.55	0.41	0.09	0.05	0.0005	*n.d.*	*<LQ*	*<LQ*	0.79	0.42	6.1	6.1	14	13	20	12	4.1	1.1	1.1	0.54
H19	2	f	4.5	65	77	0.09	*140*	0.10	37	0.15	80	*<LQ*	5.3	*<LQ*	0.034	*<LQ*	0.25	0.12	0.64	4.4	18	10	*62*	15	121	3.7	8.5	0.84	3.7
H19	2	f	11	52	154	0.53	43	0.12	0.34	*n.d.*	30	*<LQ*	1.00	*n.d.*	0.017	*<LQ*	0.11	0.07	0.27	3.9	21	8.7	*65*	19	98	5.7	11	0.40	0.70
H19	2	f	28	15	60	*<LQ*	0.09	0.10	0.10	*n.d.*	0.19	*n.d.*	*<LQ*	*n.d.*	*<LQ*	*<LQ*	*<LQ*	0.07	0.20	*<LQ*	4.7	2.5	8.6	*<LQ*	9.6	1.3	3.4	0.59	3.6
H20	2	f	4.5	93	154	0.15	*251*	0.20	4.8	0.20	108	*n.d.*	6.7	*n.d.*	0.072	*n.d.*	0.55	0.38	0.98	7.3	25	12	*70*	14	199	5.6	23	3.2	4.4
H20	2	f	11	89	212	0.63	5.1	0.12	0.43	*n.d.*	6.3	*n.d.*	0.35	*<LQ*	0.003	*n.d.*	*<LQ*	0.39	0.41	5.8	20	11	50	18	66	9.0	14	0.67	0.81
H20	2	f	28	106	150	0.85	0.28	0.27	0.10	0.18	0.32	0.08	0.08	*<LQ*	0.0010	*n.d.*	*<LQ*	0.15	0.53	7.3	11	9.2	25	20	27	12	12	1.3	4.9
H21	2	f	4.5	133	141	0.12	*213*	0.09	0.17	0.41	129	*n.d.*	4.8	*<LQ*	0.055	*<LQ*	0.63	0.38	1.6	7.6	29	16	*73*	28	188	3.2	16	2.1	1.1
H21	2	f	11	87	196	0.09	27	0.10	0.68	0.32	29	*n.d.*	0.68	0.0013	0.013	*<LQ*	0.12	0.93	0.61	7.6	29	16	*74*	17	107	3.0	18	4.4	0.59
H21	2	f	28	29	314	0.20	0.42	0.07	0.20	*n.d.*	1.7	0.05	*n.d.*	0.0007	*<LQ*	*<LQ*	*<LQ*	0.16	1.4	2.8	17	5.2	26	7.6	44	2.7	19	0.43	5.6
H23	2	m	4.5	112	120	*<LQ*	76	0.26	23	*n.d.*	38	*<LQ*	1.8	*<LQ*	0.034	*n.d.*	0.13	0.21	0.41	3.9	14	8.6	31	16	78	0.71	2.5	0.80	2.4
H23	2	m	11	40	120	0.06	0.84	0.23	0.23	*n.d.*	1.3	*<LQ*	0.66	*n.d.*	0.002	*n.d.*	*<LQ*	*n.d.*	0.15	1.8	10	3.3	16	8.7	44	0.74	0.72	0.13	0.41

H23	2	m	28	168	275	0.06	0.07	0.26	0.19	*n.d.*	0.31	1.48	0.26	*<LQ*	*<LQ*	*n.d.*	*<LQ*	0.51	0.63	6.7	8.6	12	15	39	33	1.1	1.9	3.0	3.2
H24	2	m	4.5	142	101	0.11	*87*	0.19	6.3	0.17	41	0.05	1.4	*<LQ*	0.020	*<LQ*	0.23	0.43	0.37	5.2	12	11	46	19	84	3.1	4.0	1.3	0.78
H24	2	m	11	128	60	0.74	6.1	0.23	0.11	*n.d.*	3.3	0.22	0.21	0.0006	0.003	*<LQ*	*<LQ*	0.15	0.12	7.7	7.7	16	25	56	30	2.9	2.2	0.28	0.29
H24	2	m	28	290	150	0.20	0.11	0.12	0.06	*n.d.*	0.18	0.10	0.09	0.0005	*<LQ*	*<LQ*	*<LQ*	0.42	0.11	6.8	3.7	14	8.6	22	20	5.3	2.7	1.2	0.51
P04	2	f	4.5	101	60	0.21	*119*	*n.d.*	1.2	*n.d.*	143	0.07	4.3	0.0008	0.039	*<LQ*	0.18	0.27	0.82	5.2	14	9.5	*53*	15	83	5.0	14	0.42	3.3
P04	2	f	11		18		0.13		0.16		0.96		0.08		0.0006		*<LQ*		0.33		2.5		11		*<LQ*		2.2		0.66
P04	2	f	28	61	64	0.33	0.08	*<LQ*	*<LQ*	*n.d.*	0.32	*n.d.*	0.07	*n.d.*	0.001	*<LQ*	*<LQ*	0.35	0.89	3.7	4.7	8.6	9.8	10	5.7	11	5.8	0.49	1.4
P06	2	f	4.5	214	96	0.16	164	0.21	18	0.39	205	0.09	5.6	*<LQ*	0.060	*<LQ*	0.65	1.7	1.4	13	*31*	20	*78*	24	178	16	18	3.9	1.4
P06	2	f	11	60	5	0.10	*<LQ*	0.14	*<LQ*	0.19	0.08	*n.d.*	*<LQ*	*<LQ*	*<LQ*	*n.d.*	*<LQ*	0.70	0.15	4.1	1.1	8.8	2.1	6.8	*<LQ*	6.9	1.2	0.81	0.17
P06	2	f	28	96	170	0.71	0.19	0.18	0.33	0.24	0.88	0.07	*n.d.*	0.0006	*<LQ*	*<LQ*	*<LQ*	0.72	1.8	14	9.8	24	23	24	27	18	18	0.49	3.1
P08	2	f	4.5	203	119	0.26	*135*	0.12	17	0.89	61	*n.d.*	5.4	*<LQ*	0.033	*<LQ*	0.21	0.95	1.4	14	19	17	*52*	51	127	7.5	16	3.8	4.4
P08	2	f	11	40	36	0.12	0.05	0.16	0.16	0.36	0.32	0.06	*<LQ*	*n.d.*	*<LQ*	*<LQ*	*<LQ*	0.17	0.42	4.4	4.0	6.7	8.4	41	17	2.4	1.0	0.46	1.1
P08	2	f	28	288	330	1.1	0.44	0.24	0.33	1.0	1.4	*n.d.*	*n.d.*	*n.d.*	*<LQ*	*<LQ*	*<LQ*	0.38	0.69	17	16	21	20	54	55	20	18	0.88	1.4
P09	2	f	4.5	109	35	0.29	19	0.16	1.6	*n.d.*	7.2	*<LQ*	0.72	*<LQ*	0.007	*<LQ*	*<LQ*	0.33	0.44	8.6	8.2	12	19	19	17	7.9	2.3	1.7	0.95
P09	2	f	11	33	19	0.83	0.26	0.09	0.09	*n.d.*	0.17	*<LQ*	*<LQ*	*<LQ*	*<LQ*	*<LQ*	*<LQ*	0.19	0.34	4.4	3.7	6.1	5.4	9.2	*<LQ*	4.9	2.0	0.70	0.72
P09	2	f	28	50	121	0.53	2.3	0.13	0.21	0.12	0.37	*n.d.*	0.11	*<LQ*	0.0007	*n.d.*	*<LQ*	0.30	0.56	5.8	*32*	7.2	31	7.9	25	8.2	8.4	1.2	1.8
P10	2	f	4.5	177	315	0.37	*319*	0.12	6.3	*n.d.*	136	*<LQ*	5.8	*<LQ*	0.025	*<LQ*	1.1	1.2	2.5	11	*43*	15	*116*	24	319	17	39	3.5	1.5
P10	2	f	11	33	15	0.32	*<LQ*	0.14	0.06	0.09	0.06	*<LQ*	*<LQ*	*n.d.*	*<LQ*	*<LQ*	*<LQ*	0.47	0.35	4.1	2.6	6.5	4.3	8.5	*<LQ*	5.0	1.00	0.68	0.36
P10	2	f	28	49	165	0.17	0.27	0.15	0.10	*n.d.*	0.37	*<LQ*	0.07	*<LQ*	0.0006	*<LQ*	*<LQ*	0.47	1.1	4.7	9.7	8.4	13	10	23	4.4	11	0.57	3.6
P11	2	m	4.5	82	70	0.14	147	0.15	10	0.43	136	*n.d.*	4.7	*<LQ*	0.026	*<LQ*	0.36	0.54	0.84	6.4	21	13	49	16	119	2.9	4.6	1.4	3.6
P11	2	m	11	90	20	0.16	0.08	0.10	0.07	*n.d.*	0.35	0.07	*<LQ*	*n.d.*	*<LQ*	*n.d.*	*<LQ*	0.15	0.24	4.8	2.8	11	6.3	19	*<LQ*	1.5	1.3	0.16	0.42
P11	2	m	28	163	139	0.28	0.17	0.33	0.14	0.47	0.77	*<LQ*	0.10	*n.d.*	*n.d.*	*<LQ*	*<LQ*	0.64	0.41	9.4	8.8	19	14	26	24	5.2	3.3	2.2	0.93

P12	2	m	4.5	273	172	0.25	62	0.29	6.9	0.45	24	*n.d.*	1.6	*<LQ*	0.022	*<LQ*	0.12	0.63	0.34	13	19	19	35	41	65	9.6	8.1	6.3	1.5
P12	2	m	11	137	215	0.32	0.22	0.24	0.38	0.33	0.78	0.06	*n.d.*	0.0006	*<LQ*	*<LQ*	*<LQ*	*n.d.*	0.23	12	11	17	14	42	23	0.92	7.5	0.15	1.4
P12	2	m	28																										
P13	2	f	4.5	220	214	0.30	*483*	0.16	8.0	*n.d.*	165	*n.d.*	12	*n.d.*	0.098	*<LQ*	1.7	1.0	2.5	9.6	*50*	15	*116*	30	*462*	4.7	15	0.70	1.2
P13	2	f	11	179	148	0.73	0.77	0.12	0.13	*n.d.*	*n.d.*	*<LQ*	0.08	*<LQ*	0.0005	*<LQ*	*<LQ*	0.12	1.3	8.5	13	14	19	36	33	15	10	0.39	3.1
P13	2	f	28	135	71	1.3	0.24	0.11	0.12	0.50	*n.d.*	*n.d.*	*n.d.*	*n.d.*	*<LQ*	*<LQ*	*<LQ*	0.24	0.73	11	5.5	18	8.4	46	17	32	4.1	0.94	0.46
P16	2	f	4.5	170	165	0.14	*254*	0.31	27	*n.d.*	111	*n.d.*	6.2	*n.d.*	0.044	*<LQ*	0.45	0.77	1.3	9.7	*47*	19	*96*	23	269	2.0	4.0	1.4	1.6
P16	2	f	11	206	54	0.37	0.22	0.66	0.25	*n.d.*	0.74	*n.d.*	0.07	*n.d.*	0.0009	*<LQ*	*<LQ*	1.0	0.33	14	9.5	31	19	41	19	3.9	7.5	1.0	0.40
P16	2	f	28	71	69	*<LQ*	0.40	0.10	*<LQ*	*n.d.*	0.43	*n.d.*	*<LQ*	*<LQ*	*<LQ*	*<LQ*	*<LQ*	0.45	0.20	3.8	6.3	6.4	11	6.7	40	*<LQ*	24	1.8	0.43
P18	2	f	4.5	199	129	0.16	*178*	0.10	15	0.65	55	*<LQ*	3.4	*n.d.*	0.047	*<LQ*	0.26	0.48	0.68	8.8	22	15	47	17	120	2.8	6.1	1.8	3.8
P18	2	f	11																										
P18	2	f	28																										
P19	2	f	4.5	78	85	*<LQ*	60	0.14	4.8	*n.d.*	11	0.07	1.2	*<LQ*	0.023	*<LQ*	*<LQ*	0.45	0.54	5.4	11	8.0	21	15	59	1.5	3.2	0.64	0.54
P19	2	f	11	189	262	0.10	0.17	0.15	0.35	*n.d.*	0.54	0.23	0.17	0.0006	0.0006	*<LQ*	*<LQ*	0.31	0.31	11	11	16	16	61	40	0.64	2.5	0.43	0.48
P19	2	f	28	108	114	*<LQ*	0.06	0.09	0.17	0.15	*n.d.*	0.06	0.06	*<LQ*	*<LQ*	*<LQ*	*<LQ*	0.48	0.27	5.2	5.3	8.2	8.6	22	15	1.4	1.9	0.60	0.37
P21	2	f	4.5	181	204	0.10	*306*	0.11	0.97	*n.d.*	404	0.05	4.9	*n.d.*	0.035	*<LQ*	2.3	0.20	0.37	7.7	*43*	12	*138*	19	281	5.9	14	1.6	0.73
P21	2	f	11	39	205	0.08	7.6	0.08	0.49	*n.d.*	21	*<LQ*	0.62	*<LQ*	0.003	*n.d.*	0.06	0.16	0.27	6.3	22	9.5	*62*	6.5	76	2.5	5.3	0.60	0.64
P21	2	f	28	176	196	0.14	0.12	0.16	0.20	*n.d.*	0.46	*n.d.*	0.13	*n.d.*	*n.d.*	*<LQ*	*<LQ*	0.25	0.23	8.8	7.6	13	14	24	23	3.5	3.5	0.65	0.95
P22	2	m	4.5	132	101	*<LQ*	*125*	0.23	15	0.10	47	*<LQ*	3.4	*<LQ*	0.033	*<LQ*	0.32	0.47	1.1	3.3	23	6.3	47	23	133	1.4	4.1	0.86	2.9
P22	2	m	11	44	102	*<LQ*	1.9	0.10	0.09	*n.d.*	1.8	*<LQ*	0.19	*n.d.*	0.002	*n.d.*	*<LQ*	0.24	0.52	2.7	17	4.2	27	9.8	37	1.3	3.9	0.54	0.50
P22	2	m	28	211	137	0.24	0.14	0.35	0.14	*n.d.*	0.27	*n.d.*	0.10	*<LQ*	*<LQ*	*<LQ*	*<LQ*	1.1	0.67	11	9.3	19	10	40	20	7.1	3.8	3.2	4.1
P23	2	m	4.5	147	131	0.10	76	0.43	13	*n.d.*	35	*n.d.*	5.0	*n.d.*	0.036	*n.d.*	0.09	0.79	1.5	6.5	22	10	39	26	86	4.0	5.9	0.71	3.9

P23	2	m	11		26		0.06		0.17		0.13		*<LQ*		*<LQ*		*<LQ*		0.31		3.5		7.5		*<LQ*		1.5		0.46
P23	2	m	28		80		0.21		0.14		0.08		*<LQ*		*<LQ*		*<LQ*		0.23		8.7		13		17		2.7		0.28
P24	2	f	4.5	192	148	0.06	32	0.19	14	0.72	56	0.07	4.8	*<LQ*	0.019	*<LQ*	0.23	1.3	1.5	13	20	16	*57*	11	31	6.4	8.3	3.3	2.6
P24	2	f	11	187	48	0.23	0.07	0.33	0.17	0.42	0.35	0.10	0.06	*<LQ*	*<LQ*	*<LQ*	*<LQ*	0.23	0.30	7.6	6.0	11	11	24	*<LQ*	19	5.2	0.53	0.73
P24	2	f	28	156	116	0.17	0.05	0.38	*<LQ*	0.46	0.14	*n.d.*	0.05	*<LQ*	*n.d.*	*<LQ*	*<LQ*	0.90	0.33	8.2	5.6	13	7.6	26	11	17	3.3	2.3	0.52
P25	2	m	4.5	152	122	0.16	*381*	0.17	72	0.17	248	0.06	11	*<LQ*	*0.11*	*n.d.*	1.1	0.56	2.0	7.3	*36*	11	*113*	26	280	2.7	9.4	1.1	4.8
P25	2	m	11	130	178	1.2	69	0.21	0.55	*n.d.*	52	0.13	1.2	*<LQ*	0.019	*<LQ*	0.23	0.21	0.57	8.9	*30*	12	*77*	50	113	2.6	4.8	0.21	0.63
P25	2	m	28	177	248	0.15	0.93	0.09	0.54	0.28	1.5	*<LQ*	0.24	*<LQ*	0.002	*n.d.*	*<LQ*	0.84	1.3	8.4	21	16	46	25	46	1.4	7.9	1.3	2.4
P28	2	m	4.5	350	300	0.17	*157*	0.11	1.5	*n.d.*	37	0.06	2.4	*<LQ*	0.037	*<LQ*	0.20	0.92	1.3	11	26	17	*63*	49	153	5.9	7.5	2.1	1.6
P28	2	m	11	146	97	0.19	0.13	0.14	0.11	*n.d.*	0.11	*<LQ*	*<LQ*	*n.d.*	*<LQ*	*n.d.*	*<LQ*	0.24	0.11	6.7	5.5	10	7.8	40	34	1.4	2.2	0.20	0.15
P28	2	m	28	200	287	0.09	0.18	0.08	0.08	*n.d.*	*n.d.*	*<LQ*	*<LQ*	*n.d.*	*<LQ*	*n.d.*	*<LQ*	0.27	0.86	5.4	9.3	7.9	14	33	40	0.89	6.6	0.34	3.1
P29	2	f	4.5	213	274	0.12	70	0.26	5.1	*n.d.*	87	*n.d.*	7.2	*n.d.*	0.034	*<LQ*	0.43	2.1	2.6	9.7	*39*	17	*109*	20	110	9.3	22	4.1	6.5
P29	2	f	11	12	8	*<LQ*	0.07	0.11	*<LQ*	*<LQ*	0.08	*<LQ*	*<LQ*	*n.d.*	*<LQ*	*n.d.*	*<LQ*	0.22	0.26	*<LQ*	1.3	1.6	2.2	*<LQ*	*<LQ*	1.3	1.9	0.28	0.37
P29	2	f	28	23	111	*<LQ*	0.21	0.13	0.18	0.11	0.39	*<LQ*	0.09	*n.d.*	*<LQ*	*n.d.*	*<LQ*	0.54	0.52	1.1	6.1	1.7	12	*<LQ*	24	*<LQ*	6.6	0.19	0.38
P30	2	f	4.5	287	262	0.38	*499*	0.81	78	0.43	111	*n.d.*	6.7	*n.d.*	0.097	*<LQ*	0.60	1.1	1.7	12	*36*	22	*85*	28	334	12	36	3.3	1.5
P30	2	f	11	179	40	0.37	0.27	1.2	0.13	0.56	0.40	*n.d.*	0.05	*n.d.*	0.0006	*<LQ*	*<LQ*	0.60	0.19	9.4	4.7	15	9.9	27	10	9.9	2.0	1.0	0.28
P30	2	f	28	363	165	0.47	0.16	0.59	0.19	0.61	0.26	*n.d.*	0.05	*<LQ*	*<LQ*	*<LQ*	*<LQ*	0.61	0.57	13	7.3	21	12	64	23	13	6.6	1.3	0.85
